# Interfacial Molecular Interactions as Determinants of Nanostructural Preservation in Ibuprofen-Loaded Nanoemulsions and Nanoemulsion Gels

**DOI:** 10.3390/pharmaceutics17121532

**Published:** 2025-11-28

**Authors:** Anđela Tošić, Danijela Randjelović, Branka Ivković, Ana Gledović, Tijana Stanković, Jelena Đoković, Vassiliki Papadimitriou, Tanja Ilić, Snežana D. Savić, Ivana Pantelić

**Affiliations:** 1 Department of Pharmaceutical Technology and Cosmetology, Faculty of Pharmacy, University of Belgrade, 11211 Belgrade, Serbia; andjela.tosic@pharmacy.bg.ac.rs (A.T.); ana.gledovic@pharmacy.bg.ac.rs (A.G.); tijana.stankovic@pharmacy.bg.ac.rs (T.S.); jelena.djokovic@pharmacy.bg.ac.rs (J.Đ.); tanja.ilic@pharmacy.bg.ac.rs (T.I.); snezana.savic@pharmacy.bg.ac.rs (S.D.S.); 2 Institute of Chemistry, Technology and Metallurgy, National Institute of the Republic of Serbia, University of Belgrade, 11000 Belgrade, Serbia; danijela@nanosys.ihtm.bg.ac.rs; 3 Department of Pharmaceutical Chemistry, Faculty of Pharmacy, University of Belgrade, 11211 Belgrade, Serbia; branka.ivkovic@pharmacy.bg.ac.rs; 4 Institute of Chemical Biology, National Hellenic Research Foundation, 11635 Athens, Greece; vpapa@eie.gr

**Keywords:** interfacial organization, co-solvent effect, EPI method, polyacrylate crosspolymer-6, direct gelation, nanodroplet preservation, electron paramagnetic resonance spectroscopy, atomic force microscopy

## Abstract

**Background/Objectives**: Nanoemulsions (NEs) are highly promising drug delivery systems that can be made user-friendlier by thickening to nanoemulsion gels (NEGs). However, in order to be regulatory accepted, such a transformation requires systematic understanding of the underlying interactions and stabilization mechanisms, especially when the incorporated active pharmaceutical ingredient may infiltrate the stabilizer layer. **Methods**: NEs with/without ibuprofen were submitted through direct vs. indirect gelation using three different gelling agents (carbomer 980, xanthan gum, or polyacrylate crosspolymer-6). Multi-technique characterization was employed to demonstrate nanoparticle preservation within the gel networks, a point often neglected when studying nanogels. **Results**: The nanoemulsion with the most favorable properties (55.07 ± 0.82 nm, PDI 0.075 ± 0.022) was successfully transformed into nanoemulgels with all three gelling agents, both by an indirect and direct approach. The combination of Fourier-transform infrared spectroscopy (FT-IR) and differential scanning calorimetry (DSC) revealed complex interactions and electron paramagnetic resonance spectroscopy (EPR)-discerned localization of the small-molecule model drug within the surfactants/co-solvents’ microenvironment, while atomic force microscopy (AFM) successfully visualized nanodroplets, with or without the presence of aggregates originating from the applied gelling agent. **Conclusions**: A series of complementary techniques confirmed the preservation of nanodroplets after transformation while highlighting the potential of novel polyacrylate crosspolymer-6 to produce robust gel network while effectively increasing zeta potential from −11.07 to −30.5 mV and allowing for satisfactory ibuprofen release from nanoparticles.

## 1. Introduction

Nanoformulations have been a boundless research topic recently due to their versatility and possibility to improve drug delivery through various administration routes [1,2,3]. These systems’ primary characteristic is uniform distribution of very small particles (<200 nm) that allows them to achieve better bioperformance compared to conventional dosage forms [4].

Among nanoformulations, special interest is directed towards nanoemulsions. Nanoemulsions are composed of oil and water phases with high interfacial tension between them, so the use of surfactant is required to enable effective phase joining, often with the addition of external energy. There are two main approaches to their preparation: high-energy and low-energy methods [5,6]. Although very effective, the high-energy approach requires the use of expensive, sophisticated equipment, which is why increasing attention is directed towards low-energy methods [7,8,9]. The basis of the low-energy procedure is the use of the deposited energy that the system possesses per se that originates from changes in the composition (spontaneous emulsification, emulsion phase inversion—EPI method) or properties of the stabilizer (e.g., a change in the HLB value caused by a change in temperature—phase inversion temperature—PIT method), which occurs during preparation/manufacturing [5,10].

For effective nanoemulsion preparation, a systematic understanding of the mechanisms underlying these processes is required. The production of nanoemulsions using these methods typically involves gradually adding one phase to another, leading to significant transitional changes in the system [11]. In the case of oil-in-water nanoemulsions formed by the EPI method, water is slowly added dropwise to a mixture of oil and stabilizers. At first, when a small amount of water is introduced, a water-in-oil microemulsion is formed since oil remains the dominant phase of the system. As more water is added, surfactants are hydrated, which induces the change in their curvature, and as a result, bicontinuous or lamellar structures are formed [12]. Finally, upon reaching a critical water concentration, a catastrophic phase transition occurs. At this point, surfactant molecules change their orientation, and water becomes the continuous phase in which small oil droplets are dispersed [10,13]. The properties of surfactants and the microenvironment in which they are moving play a crucial role in the successful formation of nanoemulsions through this method. Because of this, researchers have primarily studied small, highly mobile molecules such as polysorbates, sorbitan esters, and phospholipids as stabilizers of nanoemulsions produced via low-energy methods [5,14]. While the effectiveness of low-energy nanoemulsion formation is often attributed to the optimal properties of surfactants, the role of co-solvents and active pharmaceutical ingredients and their impact on microenvironment organization remains underemphasized.

In addition to enabling the successful formation of nanoemulsion droplets, stabilizers should also form a sufficiently rigid stabilizer layer that will prevent premature leakage of the encapsulated substance [15]. This is especially challenging if the encapsulated substance is a small molecule with pronounced lipophilic and hydrophilic properties, such as ibuprofen. Ibuprofen (206 Da) is attributed with very low water solubility [16]. It is a well-known NSAID, indicated for numerous diseases/conditions followed by pain and inflammation [17]. In addition to the large number of ibuprofen preparations for oral administration, (trans)dermal preparations also occupy a significant share of the market. This is understandable given that numerous diseases/conditions are accompanied by acute or chronic pain, while daily oral use of ibuprofen is associated with a high risk of gastrointestinal side effects [18]. However, there is a reasonable theoretical basis that incorporating ibuprofen within nanocarriers could achieve increased drug delivery to deeper subcutaneous structures compared to conventional topical dosage forms.

The incorporation of nanoemulsions into certain semi-solid formulations is another hot topic in the field of nanoformulations that has attracted a lot of attention from researchers [1,19,20]. Since a multitude of developed nanoemulsions are the oil-in-water type, hydrogels are certainly the most common choice for transforming liquid nanoformulations into semi-solids [21]. Nanoemulsion gels (nanoemulgels, nanogels, NEGs) are innovative carriers of a very complex structure in which nanoemulsion droplets are arranged within a gel network [22]. Their intricate structure allows them to achieve improved drug delivery to deeper structures of the skin and subcutaneous tissues compared to traditional gels. Greater substantivity and localized retention at the site of action could be an additional driving force for the nanodroplets’ penetration through the stratum corneum [23].

When transforming nanoemulsions into nanoemulsion gels, it is necessary to consider two important aspects: (i) the adequate selection of a gelling agent that will enable the formation of a sufficiently rigid 3D network in the presence of a nanoemulsion and (ii) the preservation of nanoemulsion droplets within that gel after gelation. So far, various carbomer derivatives, sodium-carboxymethylcellulose (NaCMC), and hydroxypropylmethylcellulose (HPMC) have been most extensively studied [22], but there were also reports of nanoemulsion gelation with gellan gum, fish gelatin, xanthan gum, etc. [24,25,26], each showing certain disadvantages.

In this study, three gelling agents of diverse properties were compared: carbomer 980, xanthan gum, and polyacrylate crosspolymer-6. Carbomer is a frequently used gelling agent of synthetic origin that has the ability to form a strong gel network after neutralization [27,28]. Kumara et al. examined the rheological behavior of nanoemulsion gels with different types of carbomers, among which carbomer 980 was found to be the best in modifying the rheological behavior of nanoemulsions [29]. However, recent FDA guidance for industry (*Reformulating Drug Products That Contain Carbomers Manufactured With Benzene*, FDA-2023-D-5408 of December 2023) drastically reduces the number of carbomers considered suitable for topical application, among which are carbomer 934, 934P, 974, and 1342 [30]. Xanthan gum is a cross-linked, natural derivative that, in an aqueous medium, shows gel-like behavior [31]. It is often selected not only for its natural origin but also because it does not require the neutralization step to form a gel network. Unfortunately, it provides gels with suboptimal sensory properties. Finally, an innovative polyacrylic acid derivative—polyacrylate crosspolymer-6—was introduced. Its high-performing thickening behavior, stability in a wide pH range, and compatibility with high amounts of polyhydroxyl alcohols, usually present in this type of formulation, are making this gelling agent a very interesting choice for in-depth investigation [32].

Lastly, available publications fail to provide thorough proof of satisfactory nanodroplets’ preservation after nanoemulsion gelation. This is in part due to the lack of a single suitable method that would reliably address the issue. Being aware of the importance of this characterization step, a significant part of this research study was devoted to a comprehensive examination of the internal structure of nanoemulgels using a combination of physico-chemical, thermoanalytical, and imaging techniques. Hence, one of the study’s objectives was to propose a set of complementary techniques able to provide insight into the nanodroplets’ preservation within the final, semi-solid dosage form.

## 2. Materials and Methods

### 2.1. Materials

Ibuprofen, isopropylmiristate (IPM), polysorbate 80 (Tween^®^ 80), and sorbitan oleate 80 (Span^®^ 80) were purchased from Fagron (Rotterdam, The Netherlands). Propylene glycol (Pg), polyethylene glycol (PEG) 400, sodium benzoate, trometamol, 5-doxylstearic acid (5-DSA), and 16-doxylstearic acid (16-DSA) were purchased from Sigma Aldrich (St. Louis, MO, USA). Carbomer 980 (Carbopol^®^ 980) was purchased from Lubrizol (Wickliffe, OH, USA) and xanthan gum (VANZAN^®^ NF-C) from Vanderbilt Minerals, LLC (Norwalk, CT, USA). Polyacrylate crosspolymer-6 (SEPIMAX^TM^ ZEN) was kindly donated by Seppic (Castres, France). Ultrapure water was obtained via a GenPure apparatus (TKA Wasseranfbereitungssysteme GmbH, Niederelbert, Germany).

### 2.2. Methods

#### 2.2.1. Determination of Ibuprofen Solubility in Selected Oils, Surfactants, and Co-Solvents

A solubility study was conducted using the shake-flask method in order to select the oil, surfactants, and co-solvents with the highest capacity to dissolve ibuprofen. Namely, excess amount of ibuprofen was added to 1 mL of each tested excipient, and mixture was left under moderate agitation (300 rpm) for 48 h on a vortex mixer (VELP Scientifica, Usmate Velate, Italy). The samples were then centrifuged (MiniSpin^®^ plus, Eppendorf, Hamburg, Germany) for 15 min at 3000 rpm. The concentration of ibuprofen in the tested oil and stabilizers was determined from the supernatant, diluted with a mixture of n-hexane and anhydrous ethanol, using a validated HPLC method (Section 2.2.14).

#### 2.2.2. Construction of Pseudo-Ternary Phase Diagrams

Based on the solubility results and conducted preformulation studies (for more details, see Supplementary Material), a phase behavior study was carried out in order to detect the mutual relationships and optimal concentrations of the basic components for the nanoemulsion formation. Mixtures of oil and surfactants were prepared in a range of ratios (90:10, 80:20, 70:30, 60:40, 50:50, 40:60, 30:70, 20:80, and 10:90), in which ultrapure water was added dropwise with slight manual or vortex stirring (VELP Scientifica, Usmate Velate, Italy). Any visual or textural changes were recorded, and the amount of water phase at which a low-viscous, easy-flowing liquid with a bluish sheen formed was defined as the nanoemulsion formation point. The nanoemulsion region was defined for the oil–surfactant mixtures containing polyhydroxy alcoholic co-solvents with different chain lengths, propylene glycol and polyethylene glycol 400, to study the impact of co-solvents on nanoemulsion formation.

#### 2.2.3. Sample Preparation

##### Nanoemulsion Formation Via Emulsion Phase Inversion (EPI) Method

Nanoemulsions were prepared by the low-energy method of emulsification mediated by phase inversion (EPI method). The mixture of oil, surfactants, and co-solvents was mixed for 2 min on a magnetic stirrer (IKA, Staufen, Germany) at 250 rpm. When homogenous mixture was obtained, ultrapure water was added dropwise until nanoemulsion was formed. Finally, the nanoemulsion was left on the magnetic stirrer for another 30 min in order to obtain the most uniform distribution of nanoemulsion droplets.

In the case of “active” formulations, ibuprofen was added to the mixture of oil and stabilizers and mixed for about 1 h until complete dissolution so that the final nanoemulsion contained 2% m/m ibuprofen. When deemed necessary, the process was assisted by slight heating (up to 40 °C). The nanoemulsions were stored in hermetically sealed glass vials, protected from light at 25 °C.

##### Transformation of Nanoemulsions into Nanoemulsion Gels

Nanoemulsion was transformed to nanoemulsion gels by two different approaches: direct and indirect gelation. Three different gelling agents were used: carbomer 980, xanthan gum, and polyacrylate crosspolymer-6. The nanoemulsions were thickened directly by dispersing the gelling agent by successive addition of nanoemulsion so that the final concentration of a gelling agent was 1% m/m in all nanoemulsion gels. In the case of carbomer 980-based nanogels, a 40% solution of trometamol (1.2% m/m) was used to neutralize carbomer chains. Compared to the direct approach, nanoemulsion gels were also prepared by an indirect method, whereby a pure gel matrix and the nanoemulsion were mixed in a 1:1 ratio on a magnetic stirrer at 200 rpm for about 1 h until a homogeneous mixture was achieved. All gels were preserved with sodium benzoate (0.2% m/m) that was dissolved in nanoemulsion (direct gelation method) or ultrapure water (indirect) prior to gelation. The readers should refer to [11,22] for schematic representation of the low-energy nanoemulsion preparation procedure, followed with their incorporation into gel base.

#### 2.2.4. Determination of Droplet Size, Surface Charge, and Polydispersity Index of Nanoemulsions and Nanoemulsion Gels

The average hydrodynamic droplet diameter (Z-ave) and the droplet size distribution (polydispersity index—PDI) were determined by photon correlation spectroscopy using a Zetasizer Nano ZS90 device (Malvern Instruments Ltd., Worcestershire, UK). All nanoemulsion samples were diluted with ultrapure water (1:100 *v*/*v*) before analysis in order to obtain light scattering originating only from nanodroplets.

To achieve improved delivery into the deeper structures of the skin, nanoemulsion gels should preserve small droplet size during and after gelation. Average droplet size and their distribution in the gel matrix were tested by dynamic light scattering (DLS) after dilution of each nanogel with ultrapure water (1:100 m/m). Namely, nanoemulgels were added to the defined amount of ultrapure water and left on a magnetic stirrer for 30 min at 200 rpm to completely dissolve before DLS analysis.

The same device was subsequently applied for zeta potential (ZP) monitoring as a measure of the electrophoretic droplet mobility since the surface charge of the slipping plane of nanodroplets is an important parameter that can indicate long-term stability of the created nanoemulsions. ZP is also considered as a parameter of special interest for nanogels, as it is expected that the addition of gelling agents to the system contributes to new repulsive forces and further aids dosage form stabilization.

#### 2.2.5. Electrical Conductivity

Electrical conductivity of the formulation was analyzed in order to gain insight into the internal organization of the samples. The electrode of the conductometer was directly immersed into the examined nanoemulsion samples. Measurement was conducted with Sension^TM^ + EC71 (Hach Lange GmbH, Düsseldorf, Germany), in triplicate, at 25 °C.

#### 2.2.6. pH Value

pH value of a formulation can indicate the interactions within the microstructure of both “placebo” and “active” samples, designate the suitability of the formulation for the intended route of administration, and also predict the dissolved/undissolved fraction of the drug in the formulation that can affect its skin permeation. The electrode of the pH meter pH80 + DHS (XS instruments, Peveragno, Italy) was directly immersed in the tested nanoemulsions and nanogels in triplicate at 25 °C.

#### 2.2.7. Stability Assessment

To test preservation of the internal structure of nanoemulsions with and without ibuprofen, a basic physico-chemical characterization was carried out during three months of storage. Nanoemulsion samples were visually examined for any indications of phase separation or the formation of a (nano)emulsion ring on the glass tube. Small amounts of nanoemulsion gels were placed on microscopic plates in thin layers and observed for homogeneity, transparency, and color. Particle size, PDI, zeta potential, pH value, and electrical conductivity were measured and compared with initial findings for all the investigated samples.

#### 2.2.8. Fourier-Transform Infrared Spectroscopy (FT-IR)

The ibuprofen loaded-nanoemulsions and nanogels, corresponding placebo formulations, pure ibuprofen, and all excipients involved were analyzed using a Nicolet iS10 FT-IR spectrometer (Thermo Fisher Scientific, Loughborough, UK) in order to study complex interactions between the active and other formulation ingredients and confirm ibuprofen entrapment into nanodroplets. Additionally, FT-IR analysis was conducted in order to investigate the complex interactions between nanoemulsions and gel matrix and to preliminarily confirm preservation of nanosized droplets in the formed gels.

#### 2.2.9. Differential Scanning Calorimetry (DSC)

Thermal analysis was carried out on a DSC 1 device (Mettler-Toledo GmbH, Greifensee, Switzerland) in order to study the physical state of ibuprofen in the developed nanoemulsions and nanogels as well to confirm the existence of interactions between the samples’ components gained via FT-IR analysis. The thermal behavior of the nanoemulsion gels obtained by direct and indirect gelation was tested in order to investigate the microstructure differences between gels obtained by two different approaches.

Approximately 5 mg of each sample were put into hermetically sealed aluminum pans and heated under continuous nitrogen flow from 25 °C to 150 °C at a rate of 5 K/min, while an empty pan served as a control. Relevant thermoanalytical parameters were analyzed with Mettler Toledo STARe program.

#### 2.2.10. Encapsulation Efficacy Study

The test has been carried out by the ultrafiltration method using centrifugal filters (Amicon Ultra-4; MWCO 10 kDa, Millipore, Barueri, Brazil). Samples were centrifuged at 4500 rcf for 90 min, after which the filtrates were collected and ibuprofen content was determined. Encapsulation efficiency (EE%) is expressed as a ratio of the total amount of ibuprofen and the amount present in the filtrate after exposure of the sample to centrifugal force.

#### 2.2.11. Electron Paramagnetic Resonance (EPR) Spectroscopy

This investigation was conducted using a Bruker EMX EPR spectrometer (Bruker, Billerica, MA, USA) with X-rays and a WG-813 Q-Wilmand flat cell (Buena, NJ, USA). As spin probes, 5-doxylstearic acid (5-DSA) and 16-doxylstearic acid (16-DSA) were used, which at defined positions have an unpaired electron originating from the nitroxide ring. Doxylstearic acid is an amphiphilic molecule that, when mixed with nanoemulsions, stations itself on the water–oil interface. Addition of nitroxide ring in the position C5 of this molecule describes environment of surfactant layer near the water phase, while derivatization with a nitroxide ring at position C16 describes environment of surfactant layer in proximity of the oil phase. The organization and rigidity of the nanoemulsion’s stabilizer layer in the two mentioned important positions (near the water or oil phase), with or without ibuprofen, were monitored by tracking the mobility of this ring, which is represented by the rotational correlation time (τR), the order parameter of the spin probe position (S), and isotropic hyperfine coupling constant (αN).

#### 2.2.12. Atomic Force Microscopy (AFM)

Droplet size of the nanoemulsions and their morphology were examined with the NTEGRA Prima Atomic Force Microscope (NT-MDT, Moscow, Russia). Intermittent-contact AFM mode was chosen as optimal for this type of sample, and NSG01/Pt silicon cantilever was used. This device was also used for investigating the 3D matrix organization of the nanoemulsion gels, nanodroplet preservation, and their localization within the gel matrix. Visualization and analysis of the obtained AFM data were performed using Gwyddion 2.61 (free and open-source software, Czech Metrology Institute) software. In order to form a sufficiently thin dried sample suitable for AFM analysis, sample preparation differed according to samples’ consistency (liquid or semi-solid). Prior to analysis, nanoemulsions were diluted in ultrapure water (1:500, *v*/*v*), while in the case of nanoemulgels, significantly greater dilution was required (1:2000 *v*/*v*). After dilution, 10 µL of each sample were placed on mica discs (Highest Grade V1 AFM Mica Discs, Ted Pella Inc., Redding, CA, USA) and dried in a vacuum dryer for 2 h at 25 °C.

#### 2.2.13. Rheological Behavior

A rotational rheometer (Rheolab MC 120, Paar Phisica, Ostfildern, Germany) equipped with a cone/plate measuring system (diameter 50 mm, angle 1°) was used to test the rheological behavior of nanoemulsion gels obtained by direct vs. indirect gelation method. The analysis was carried out under continuous shear conditions at 20 ± 0.1 °C, 72 h to 96 h after nanoemulgels’ preparation in order to guarantee full internal structure formation. Shear rate was increased from 0 to 200 s^−1^ in the first phase and decreased from 200 to 0 s^−1^ in the second phase of the test. A comprehensive understanding of the flow behavior and structural features of the formulations was provided by the evaluation of critical rheological parameters such as flow profiles, maximum and minimum apparent viscosities, and hysteresis areas.

#### 2.2.14. In Vitro Release Testing (IVRT) of Nanoformulations

In vitro release of ibuprofen from prepared formulations was tested using vertical Franz diffusion cells (n = 6, Gauer Glas, D-Püttlingen, Püttlingen, Germany) under infinite dose conditions to ensure a continuous driving force for ibuprofen diffusion. Polycarbonate membranes (Nuclepore™, Whatman, Maidstone, UK) were used as a barrier for diffusion. Membranes were immersed in phosphate buffer solution pH = 7.4 (PBS) for 1 h prior to the study in order to activate and hydrate them. Receptor chambers were also filled with PBS as the receptor medium, up to 12 mL. Then, previously activated polycarbonate membranes were placed on the top of the receptor chamber and connected with donor chambers. The connected Franz cells were placed in a water bath preheated to 32 °C and left for another 1 h to heat up the receptor medium. Nanoemulsion gels and nanoemulsion (approximately 300 mg) were placed in the donor chamber and covered with an occlusive film (PARAFILM^®^ M, Amcor Flexibles North, Neenah, WI, USA) to prevent evaporation. Samples (600 µL) were collected at predefined time intervals (after 1, 2, 4, 6, 8, 22, and 24 h), and after each sampling, the same volume of the receptor medium was added to the receptor chamber in order to maintain sink conditions. The amount of ibuprofen in the collected samples was determined with a validated high-performance liquid chromatography (HPLC) method.

#### 2.2.15. Ibuprofen Quantification

The quantitative determination of ibuprofen in pharmaceutical formulations and during the IVRT was performed using an HPLC method. The analysis was conducted on a Dionex Ultimate 3000 system equipped with a diode array detector (DAD) and a Zorbax Eclipse Plus C18 analytical column (5 µm, 150 × 4.6 mm). The column temperature was maintained at 30 °C. The mobile phase consisted of 0.1% (*v*/*v*) phosphoric acid and acetonitrile mixed in a 35:65 (*v*/*v*) ratio, delivered at a constant flow rate of 1.2 mL/min. The detection wavelength was set at 220 nm, corresponding to the absorption maximum of ibuprofen.

#### 2.2.16. Data Analysis

Whenever possible, results were presented as mean value ± standard deviation of at least three measurements. Statistical analysis was performed using software PASW Statistics 18.0 (SPSS Inc., Chicago, IL, USA). After checking whether the data followed a normal distribution, Student’s *t*-test or ANOVA with Tukey post hoc were used, depending on the number of groups compared. Statistical significance was considered for a *p*-value < 0.05.

## 3. Results and Discussion

### 3.1. Characterization of Interfacial Properties Governing Nanoemulsions’ Performance

For the production of nanoemulsions in this study, emulsion phase inversion has been chosen as an easy, reproducible approach, not requiring expensive equipment [33]. EPI happens when a critical amount of water is titrated in the mixture of oil and hydrophilic surfactant(s) [34]. With the addition of water, the system passes through different transition phases until oil/water nanoemulsion with very small droplets is formed [10,35]. Since low-energy nanoemulsion fabrication does not require sophisticated equipment, the energy that is needed for the nanoemulsion formation is provided by the system per se. Hence, the right selection of oil, surfactants, and co-solvents is a critical step during the formulation of these carriers [5].

#### 3.1.1. Selection of Nanoemulsion Components

The solubility of ibuprofen was tested in the oils of different chain lengths: isopropyl myristate (IPM), medium-chain triglycerides (MCT), decyl oleate, and octyldodecanol, as well as in low molecular weight surfactants and co-solvents: polysorbate 80, polysorbate 60, ethanol, propylene glycol (Pg), and polyethylene glycol 400 (PEG 400). This preformulation study (Table 1) was conducted in order to discern oils, surfactants, and co-solvents with the highest ibuprofen solubility and to maximize the amount of active that could be fully dissolved in the nanocarrier.

IPM was selected as the oily phase of the nanoemulsion since, among other tested oils, it has shown the highest capacity to dissolve ibuprofen (saturation solubility 65.94 ± 0.51 mg/mL). Polysorbates were chosen because they are small, skin-friendly molecules, with notable mobility, i.e., for the properties that generally make them suitable stabilizers for low-energy nanoemulsions [36]. Although both tested polysorbates, Tween 80 and Tween 60, have shown satisfactory ibuprofen solubility (203.65 ± 1.32 mg/mL and 166.63 ± 0.95 mg/mL, respectively), Tween 80 was selected for the future investigation due to higher ibuprofen solubility.

Co-solvents have an important role during nanoemulsion formation via low-energy methods since they have the ability to affect the surfactants behavior on the oil–water interface by changing the optimum curvature, critical micellar concentration, and concentration of surfactant needed for nanoemulsion production [5]. Although ethanol showed the highest saturation solubility of ibuprofen (337.78 ± 0.81 mg/mL) compared to the other tested co-solvents, it was excluded from further investigation for the possibility of contributing to skin irritation, especially in combination with relatively high surfactant concentration, which is a standard requirement in the low energy methods [37,38]. On the other hand, Pg and PEG 400 have both shown satisfactory saturation solubility of ibuprofen. Additionally, Pg and PEG 400 are able to introduce other functions to the formulation, such as that of humectants, which is why both co-solvents were chosen for further research.

#### 3.1.2. Phase Behavior of Nanoemulsions

**Figure 1:**
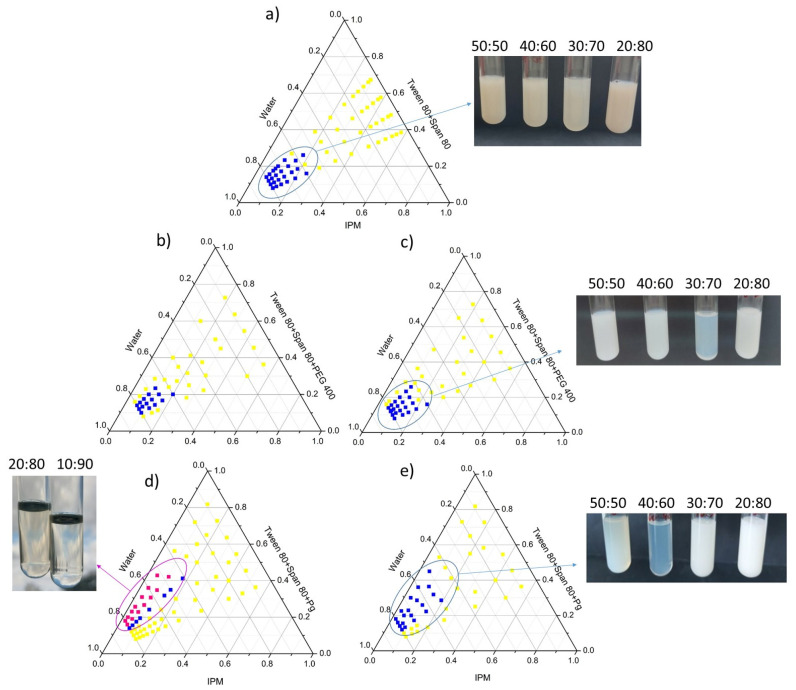
Pseudo-ternary phase diagrams obtained with different mixtures of stabilizers: (**a**) Tween 80 + Span 80 in 3:1 ratio; (**b**) Tween 80 + Span 80/PEG 400 (S/CoS ratio 1:1); (**c**) Tween 80 + Span 80/PEG 400 (S/CoS ratio 2:1); (**d**) Tween 80 + Span 80/Pg (S/CoS ratio 1:1); and (**e**) Tween 80 + Span 80/Pg (S/CoS ratio 2:1) with corresponding (nano)formulation appearance in glass tubes and the precise ratio of excipients within. Notes: yellow spots—unstable transition phases; blue spots—nanoemulsion region; pink spots—microemulsion region.

The optimal oil/surfactant mixture/water ratio is another critical step during nanoemulsion formation with the low-energy method. Nanoemulsion phase behavior was investigated for different oil and surfactant mixtures, and as a result, pseudo-ternary phase diagrams were constructed. The nanoemulsion region was marked as represented in Figure 1.

The phase behavior of the Tween 80/Span 80 mixture with an HLB value of 12 was further investigated since our preformulation study (Supplementary Material, Table S1) implied that HLB around 12 provides a blend with optimal IPM emulsification capability. As represented in Figure 1a, the Tween 80/Span 80 mixture provides successful nanoemulsion formation with a surfactant-to-oil ratio (SOR) between 1.0 and 2.5. As co-solvents are also bound to exert a considerable influence on the nanoemulsion fabrication, the two polyhydroxyl alcohols (Pg and PEG 400) were subsequently introduced to the mixture, and their impact on the phase behavior was investigated. Figure 1b,d clearly show that the addition of either PEG 400 or Pg to the ternary system with the surfactant/co-solvent (S/CoS) ratio of 1:1 provides a more constrained nanoemulsion region, indicating that the addition of co-solvents in this ratio creates an unfavorable environment for the nanoemulsion formation. Interestingly, the addition of Pg in the S/CoS 1:1 ratio favorized the formation of microemulsion (Figure 1d). On the other hand, extended or equivalent nanoemulsion regions were observed with the addition of PEG 400 (Figure 1c) and Pg (Figure 1e) in the S/CoS 2:1 ratio, respectively, resulting with more bluish, transparent, and easy-flowing nanoemulsions compared to the initial Tween 80/Span 80 mixture.

Finally, from the pseudo-ternary phase diagrams, three different formulations were selected with fixed amounts of the co-solvents. In each of them, ibuprofen (2% m/m) has been incorporated (i.e., active, a-labelled samples). Their qualitative and quantitative composition is represented in Table 2.

#### 3.1.3. Physico-Chemical Properties of Placebo and Ibuprofen-Loaded Nanoemulsions

The influence of selected co-solvents (PEG 400, Pg, or their combination) on the interfacial layer’s organization and the main physico-chemical properties of nanoemulsions have subsequently been studied (results are represented in Table 3). Average droplet size for all three placebo formulations was in the nanorange (<200 nm), confirming successful nanoemulsion preparation via the EPI method. The lowest particle size was observed for the formulation NE_F1, which contained the combination of two co-solvents with different chain lengths, indicating that the intertwining of their hydrophilic chains produces the boundary environment in which surfactant molecules optimally position themselves, forming oil droplets of the smallest diameter (80.56 ± 0.41 nm). As noted earlier, the co-solvent(s) may additionally change the physico-chemical properties of the water phase, such as density, viscosity, and refractive index. Here, the increase in the water phase viscosity affected by the addition of co-solvents (inherent viscosities ~50 and ~100 mPa·s for Pg and PEG 400, respectively) has favorably altered the mass transport kinetics of surfactant and oil molecules at the phase boundary, resulting in the formation of smaller oil droplets [39].

Polydispersity index was in an acceptable range for almost all placebo formulations (≤0.25), excluding the formulation NE_F3. Formulations that contain just Pg as the co-solvent produced nanoemulsions with the lowest PDI (i.e., 0.184) compared to the formulations that contain PEG 400, for which the PDI value was above 0.2. These results could also be explained by different viscosities of the two co-solvents, but more importantly by their different ability to form hydrogen bonds with water, ultimately decreasing the interaction of water with the oily phase [40].

Bearing in mind the fact that nanoemulsion stabilization was enabled by Tween 80/Span 80 combination, zeta potential values expectedly ranged between −26.7 and −14.1 mV. Namely, zeta potential is a measurement of electrophoretic mobility on the slipping plane of nanoemulsions, and the zeta potential around −20 mV is a satisfactory result for the nanoemulsions prepared solely by non-ionic surfactants [41]. Non-ionic surfactants stabilize nanoemulsions predominantly through steric protection provided by hydration of their polymer chains. Because of that, the principal non-ionic surfactant, such as Tween 80, should be combined with a compatible co-surfactant (i.e., Span 80), resulting in a densely packed stabilizing layer with somewhat lower ZP values [4]. Considering that all three nanoemulsions are attributed with pH values in a slightly acidic range, the observed negative zeta potential probably originates from the attracted OH^−^ ions from the water phase in combination with the steric effect of the polymeric stabilizers [42]. Differences in zeta potential between formulations with different co-solvents can be explained by the fact that Pg, PEG 400, and their combination make specific environments close to the interfacial layer, resulting in different amounts of absorbed OH^−^ ions on the slipping plane. The highest magnitude of ZP was observed for the formulation NE_F2 (−26.7 mV), while the lowest magnitude of ZP for NE_F1 (−14.1 mV), indicating that the combination of PEG 400 and Pg produces density-vise, the most complex environment. This barrier hinders the OH^−^ ions to be absorbed on the interfacial layer.

Ibuprofen-loaded nanoemulsions were transparent, easy-flowing liquids without visual signs of instability, indicating successful incorporation of the amphiphilic active to the fragile low-energy nanoemulsion carrier. The incorporation of ibuprofen to the nanosystem inevitably led to significant changes (*p* < 0.05) in all tested parameters compared to placebo formulations.

The addition of ibuprofen to the nanoemulsions resulted in a notable decrease in average droplet size (Z-ave was in the range 54.35−60.38 nm), with a significant decrease in PDI value. The very low PDI value of the formulation NE_F1a (0.075 ± 0.022) indicated ibuprofen’s ability to affect the nanodroplet formation, resulting in an almost monodisperse system. The magnitude of zeta potential also decreased, suggesting that ibuprofen changed the organization of molecules in the water–oil interface, resulting in lower absolute ZP values. The decrease in the aforementioned parameters was in correlation with the decrease in pH. Ibuprofen is a derivative of propionic acid with a pKa value of 4.4 [43]. The addition of such a weak acid to the formulation resulted in a shift in the equilibrium between the concentrations of free OH^−^ and H^+^ ions in favor of H^+^ ions. As a result, fewer free OH^−^ ions were attracted to non-ionic surfactants, which was reflected in a significant decrease in the absolute ZP value for all ibuprofen-loaded formulations compared to placebo ones. On the other hand, negatively charged COO^−^ ions could theoretically increase the magnitude of ZP, but it should be kept in mind that other complex interactions occur on the slipping plane. This result suggests that ibuprofen possibly changed the organization of the stabilizer layer. Its presence affects the interface by changing the organization of the surfactant layer, altering its orientation and density, thereby partially masking the surface charge. On the other hand, this effect is least pronounced in the NE_F1a formulation compared to the placebo counterpart, which further confirms that different microenvironments caused by the varying co-solvents affect the overall organization of the slipping plane. The assumption that ibuprofen changed the organization of the interfacial layer is also confirmed by the fact that there was a significant change in electrical conductivity between the placebo and active formulations.

#### 3.1.4. Investigation of Interfacial Organization

Given that the incorporation of ibuprofen into nanoemulsions contributed to a significant reduction in droplet size, PDI, and ZP, additional studies were conducted in order to investigate the interfacial organization in more detail and determine where ibuprofen is located and how it alters the organization of the interfacial layer. In order to describe these singularities, electron paramagnetic resonance (EPR) spectroscopy, FT-IR, and DSC analysis were subsequently conducted.

EPR analysis has shown subtle differences in the surfactant layer microenvironment between formulations (Table 4, supported by Figure S1 within the Supplementary Material).

EPR analysis is a sophisticated technique with the ability to describe the rigidity, organization, and microstructure of a surfactant monolayer at the oil–water interface [44]. The rotational correlation time (τR) represents the rotation speed of the probe’s nitroxide ring and thus indicates the rigidity of the surfactant monolayer, while the parameter S describes the order degree of the microenvironment near the nitroxide ring and could vary from 0 to 1. For a full understanding of the polarity, rigidity, and organization of the stabilization layer, these two parameters should be interpreted simultaneously [45]. The magnitude of the isotropic hyperfine coupling constant (αN), which is directly correlated with the distance between peaks in the EPR spectra, reveals the degree of spin polarization and delocalization of the unpaired electron [46]. In the present study, it was specifically interesting to compare αN constants in the microenvironment near the 5-DSA probe in order to study the impact of present co-solvents on the surfactant monolayer organization.

Analyzing the EPR results for placebo formulations, it was noticed that the part of the monolayer near the 5-DSA probe showed more compactness and rigidity than the deeper parts, analyzed with the 16-DSA probe. Namely, the rotational correlation time (τR) obtained with the 5-DSA probe was similar for all three formulations (being in the range 2.11−2.13 ns), which was 5 times higher than the results obtained with the 16-DSA probe (where τR ranged between 0.32 and 0.40). The order parameter (S) additionally confirmed this state since it showed higher values with 5-DSA than with the 16-DSA probe. The isotropic hyperfine coupling constant was the smallest for the sample NE_F2 relative to the other two placebo formulations, suggesting that Pg affects the polarity and flexibility of the microenvironment near the unpaired electron, weakening its interaction with the nucleus. The combination of the two co-solvents in the sample NE_F1 resulted in the highest value of this parameter, indicating that their combination stabilizes the microenvironment and strengthens the hyperfine interaction of the unpaired electron with nitrogen. An EPR analysis demonstrated that the selected co-solvents noticeably contributed to greater compactness of the stabilizing layer by building different types of interactions with surfactant molecules, affecting their arrangement on the monolayer.

The incorporation of ibuprofen into nanoemulsions affected the organization of the oil–water interface. In all three ibuprofen-loaded formulations, a decrease in the τR value was observed, indicating that the spin probe rotated faster due to a decrease in the rigidity of the stabilizer layer. Formulation NE_F1a showed a modest decrease in the τR value and an increase in the order parameter S with the addition of ibuprofen, confirming that ibuprofen contributed to better organization of the oil–water interface. The lowest rigidity of the stabilization layer facing the aqueous phase was observed for the formulation NE_F2a, suggesting that ibuprofen infringes on the surfactant organization to the highest degree in the formulation that solely contained Pg as the co-solvent. The surfactant layer near the oil phase also underwent notable changes with ibuprofen addition. As opposed to the layer closer to the aqueous phase, this part of the stabilization layer showed higher compactness and rigidity with the addition of ibuprofen compared to placebo formulations (the τR value was higher in all formulations except the NE_F1a, where a small decrease in this parameter was observed).

EPR pointed out that ibuprofen is mainly situated within the surfactant monolayer near the aqueous phase, thus affecting the organization of the outer ring of the stabilization layer. Considering the structure of this small molecule and the fact that the highest saturation solubility of ibuprofen was observed in Tween 80 (203.64 mg/mL) and PEG 400 (239.75 mg/mL), its positioning in this part of the nanocarrier can be considered a reasonable finding. Also, this analysis provided additional proof that the combination of Pg and PEG 400 resulted in the nanoemulsion with the highest compactness and rigidity of the stabilizer layer, which was affected by the addition of ibuprofen.

The claim that the selected stabilizer mixture resulted in formulations with a compact stabilizer layer was confirmed by testing the encapsulation efficiency. All three formulations showed very high encapsulation efficacy (99.46–99.68%) after ultrafiltration, confirming that ibuprofen was completely incorporated within the nanoemulsion droplet.

Further confirmation of ibuprofen localization and its exact physical form in the nanoemulsion was provided by the FT-IR and DSC results.

**Figure 2:**
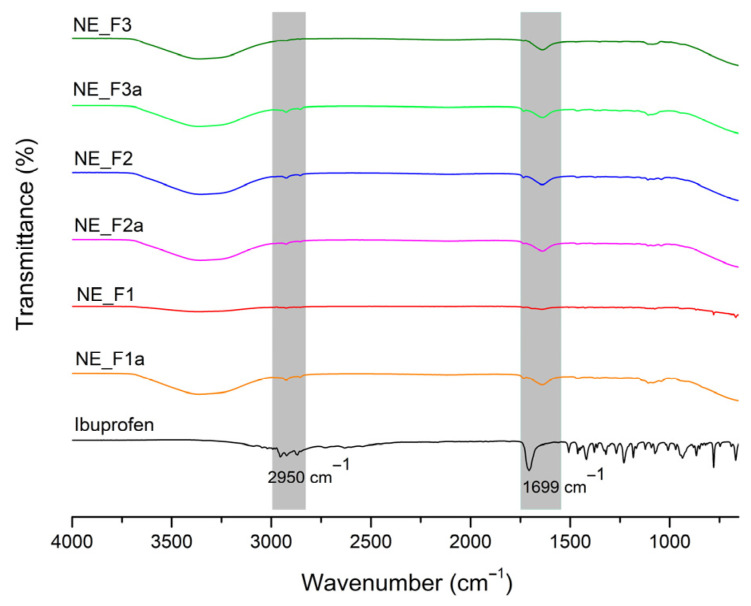
FT-IR spectra for pure ibuprofen, placebo, and ibuprofen-loaded nanoemulsions. Gray-shaded regions mark parts of the spectrum with characteristic ibuprofen peaks.

Ibuprofen absorbs the infrared spectrum, generating distinctive peaks between 2950 and 2870 cm^−1^ (vibration of aliphatic C-H bond) and at 1699 cm^−1^ (stretching of carbonyl group’s C=O) and numerous peaks in the lower part of the spectrum (vibration of the methyl CH_3_ group, stretching and deformations of aromatic C=C bonds) [47,48]. All particular peaks at the mentioned wavelengths (Figure 2) were completely quenched or reduced in the spectrum derived from the active samples, suggesting that ibuprofen is completely dispersed in all three nanoformulations. The characteristic peak originating from C=O stretching was found to be significantly decreased and shifted from 1699 cm^−1^ to 1637 cm^−1^ in the spectrum obtained for the sample NE_F1a. This shift directly verifies ibuprofen incorporation inside of the carrier and its binding by the formation of the hydrogen bonds between the stabilizer molecules (the most pronounced interactions were observed with Tween 80 and PEG 400; see Figure S2 within the Supplementary Material) and the free pair of oxygen electrons from the carboxyl group of ibuprofen, which reduces the vibrations of this group and quenches the characteristic peak. A significant reduction of the peak between 2900 and 2870 cm^−1^ was also observed, which may indicate the positioning of ibuprofen within the oil droplet, but it is difficult to interpret with complete certainty since both IPM and stabilizers provide peaks at similar wavelengths (Supplementary Material, Figure S3). Also, it is important to emphasize the complete disappearance of the high peak frequency in the lower part of the spectrum, confirming previous findings about ibuprofen’s complete incorporation into nanoemulsions. Co-solvent(s) effects that were specifically examined in this study could be observed in the form of a broad peak band between 3500 and 3000 cm^−1^ in all tested formulations, both placebo and active ones. This wide band probably depicts the stretching and bending O–H vibrations originating from co-solvents since broad peaks at these wavelengths were observed in the spectra originating from pure propylene glycol and PEG 400 (Figure S3), confirming once again that co-solvents affect the microenvironment near the stabilizing monolayer and thus determine the formation of nanodroplets. Overall, FT-IR analysis supported our initial results and demonstrated that ibuprofen is evenly distributed throughout the nanoemulsion system via the formation of hydrogen bonds between the carboxyl group’s free electron pair and hydrogen molecules from surfactants and co-solvents. The significance of co-solvents’ proper selection for the successful formation and stabilization of low-energy nanoemulsions with ibuprofen was brought to light by the FT-IR analysis.

**Figure 3:**
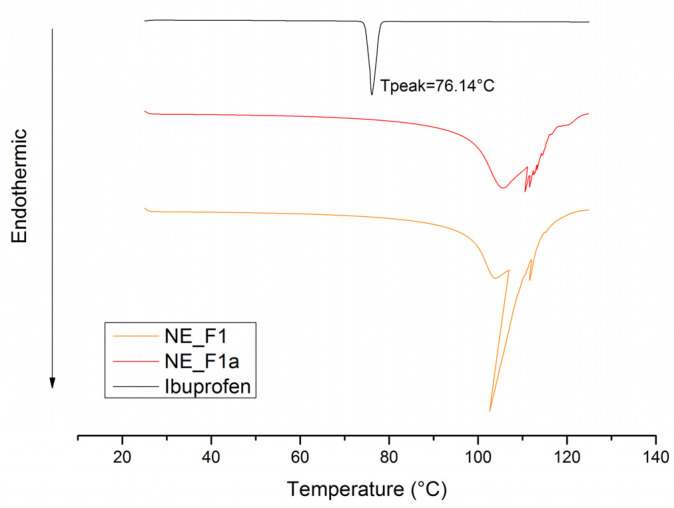
DSC thermograms for pure ibuprofen, placebo (NE_F1), and ibuprofen-loaded nanoemulsion (NE_F1a).

DSC is a powerful thermosensitive method commonly used to reveal the physical state of the incorporated active pharmaceutical ingredient. However, it can also complement information provided by other techniques concerning a drug’s effect on the formulation’s microstructure. Ibuprofen is a crystalline substance that exhibits a sharp endothermic peak at about 76 °C, which is previously well described in the literature [49]. The fact that this characteristic peak cannot be observed in the thermogram obtained for the active NE samples (such as the NE_F1a scan given in Figure 3), confirms the homogeneous incorporation of ibuprofen within the nanoemulsion. Interestingly, the thermogram obtained for NE_F1 exhibits a very sharp peak around 105 °C, which probably originates from the release of bound water that was incorporated within the complex surfactant/co-surfactant-mediated layers. Similar thermograms are obtained for other nanoemulsions as well (see also Figures S4 and S5 in Supplementary Material). In the case of the active formulations, the peaking is equally complex but notably less sharp, suggesting that ibuprofen’s interaction with stabilizers resulted in a smaller amount of bound water within the oil–water interface.

#### 3.1.5. Stability Study

**Figure 4:**
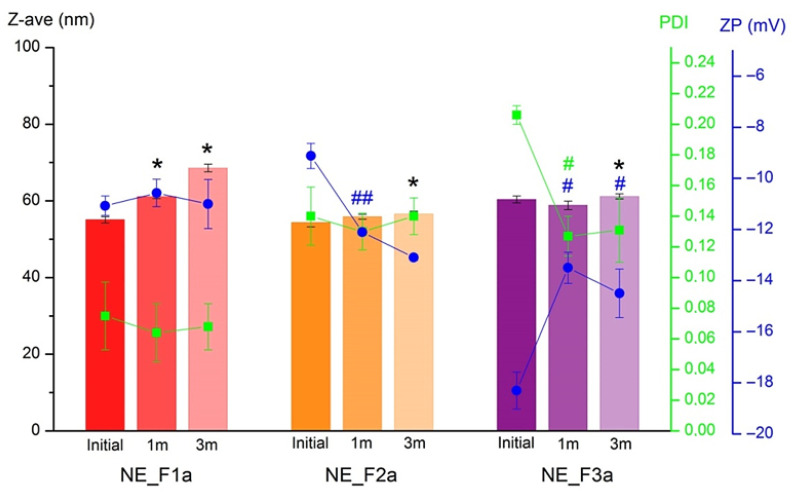
Stability of ibuprofen-loaded nanoemulsions after 3 months of storage (* *p* < 0.05, # *p* < 0.01, ## *p* < 0.001 compared to initial measurments.)

After three months of storage at room temperature, there were no signs of physical instability in any of the monitored samples. None of the formulations showed signs of phase separation, the presence of undissolved ibuprofen crystals, or the so-called (nano)emulsion rings on the glass vials used for storage. Good stability was also confirmed by monitoring the basic physico-chemical parameters shown in Figure 4. Although a significant increase in particle size after three months of storage was observed (*p* < 0.05) in all three nanoemulsions, average droplet size was still very low and nanoscaled (<70 nm). A change in the PDI was observed only in the sample NE_F3a, and the decrease in this parameter probably originated from the additional stabilization of the nanosystem during the storage period. All three formulations showed excellent robustness to centrifugation and dilution (Table S2). Although all three formulations showed satisfactory stability in the monitored parameters, taking into consideration all the collected results, the formulation NE_F1a was submitted through gelation in the next phase of the study.

### 3.2. Multi-Technique Characterization of Nanoparticle Preservation upon Nanoemulsion-to-Nanogel Transformation

**Figure 5:**
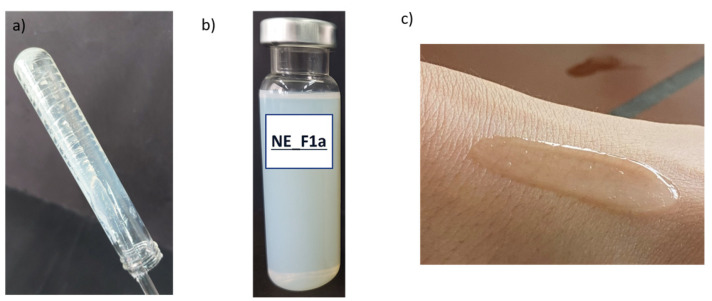
Different stages during formulation preparation: (**a**) initially observed thick transition phase resembling honey’s consistency, (**b**) o/w nanoemulsion formation after the addition of critical amount of water, and (**c**) transformation into nanoemulsion gel by direct gelation of NE with polyacrylate crosspolymer-6.

Nanoemulsion was successfully transformed into nanoemulsion gels using both a direct and indirect approach (see Supplementary Material, Figure S6). Interestingly, the sensory properties of nanoemulgels depended on the used preparation method. Namely, gels obtained with the direct approach were more viscous and with a denser tactile feel, while the indirect approach resulted in more liquid, easy-to-flow samples. Although the gels obtained with xanthan gum were more transparent compared to gels prepared with the other two gelling agents, all nanogels were homogeneous, with a bluish shine, which originates from the nanoscale structures contained within the gel network (Figure 5).

#### 3.2.1. Physico-Chemical Properties of Nanoemulsion Gels

The specific aim of this study was to compare the potential of three different gelling agents for the transformation of a nanoemulsion into a more user-friendly nanoemulsion gel. In addition to the traditional gelling agents (carbomer 980 and xanthan gum), the potential of an innovative polyacrylic acid derivative—polyacrylate crosspolymer-6—has also been tested. The main physico-chemical properties of nanoemulsion gels obtained with different gelling agents by two preparation methods are presented in Table 5.

The most important property of nanoemulsion gels is the preservation of nanodroplets inside the gel network after gelation. The formulation quality, improved drug delivery and subsequently desired effects at the site of action directly depend on it [50]. However, transformation to a semi-solid form can trigger destabilization of already fragile nanostructures [21]. Therefore, testing the inertness of gelling agents in order to create a gel network in the presence of nanoemulsions without causing structural disruption is a critical step during the development of nanoemulsion gels. In this work, the influence of the preparation method on the preservation of nanoemulsion droplets in the gel network was additionally examined. Although the direct approach would be preferred in manufacturing settings, it may prove to be more aggressive towards the nanoemulsion due to the fact that the gelling agent draws the nanoemulsion’s water phase for its hydration, meanwhile expanding its polymer chains.

All tested nanoemulsion gels showed very small particle sizes for both preparation approaches (<100 nm). Particle size was in the range between 60.65 nm and 71.63 nm in almost all tested nanoemulsion gels, which was slightly higher compared to particle size obtained for the pure nanoemulsion (55.07 ± 0.82 nm). The highest particle size has been recorded for the formulation NEG_C980_d (94.03 ± 0.63 nm), suggesting that direct gelation of nanoemulsions with carbomer 980 results in a slight impairment of nanostructure. On the other hand, indirect gelation with carbomer 980 resulted in a significantly lower particle size (*p* < 0.001). Significant differences between two preparation methods are noticed for the polyacrylate crosspolymer-6 as well, suggesting that droplets of slightly larger diameter are obtained when the gel is prepared by the direct method. The preparation method failed to affect the particle size only in the case of nanoemulsion gels with xanthan gum since no significant differences in nanodroplets have been noticed between directly and indirectly gelled nanoemulsions. Expectedly, the transformation of the liquid to the semi-solid state has slightly increased the PDI values in all tested nanogels. The increase in PDI is directly related to the introduction of polymers into the system since polymer self-assembly can seldom be prevented [51,52]. However, it is important to emphasize that thickening did not lead to the disruption of the nanoemulsion structure and that PDI values still remained highly acceptable (<0.25).

The transformation of nanoemulsions to nanoemulsion gels resulted in a significant increase in the magnitude of the zeta potential (*p* < 0.01) in all tested samples compared to the pure nanoemulsion. This is due to the creation of new interactions between the hydrated molecules of gelling agents and nanoemulsion stabilizers. Namely, in the case of all three tested gelling agents, there was an increase in negative charge on the slipping plane of the nanoemulsion, which was expected given that all three gelling agents belong to the group of anionic polymers [28,53]. It is assumed that during the gelation of nanoemulsions, hydrated polymer molecules adhered to the surface of the oil droplet, resulting in the addition of a significant negative charge on the measured slipping plane. The negative repulsion forces are certainly also intensified by the additional steric effect, which probably creates a denser stabilization layer around the oil droplet, overall increasing the repulsion of the nanoemulsion droplets and contributing to better stabilization after transformation. This result was in accordance with reports from the literature where the addition of carbomer 980 and xanthan gum contribute to the increase in absolute ZP values [54,55]. The highest magnitude of the ZP was noticed in the formulation NEG_SZ_d (−30.5 ± 0.9 mV), which was three times higher than that of the corresponding nanoemulsion (−11.07 ± 0.38 mV), suggesting that specific structure of the polyacrylate crosspolymer-6 has the biggest potential to contribute to the electrostatic repulsion of the droplet within the gel network. The pH value of the formulation, which in the case of all gels was in the skin-acceptable range of 5.46–5.72, certainly contributes to the overall ion balance and favors the dissociation of gelling agents and the creation of a negative ionic charge on the surface of the nanoemulsion droplets.

#### 3.2.2. Visualization of Nanoemulsion and Nanoemulsion Gels’ Structure

DLS analysis, although powerful as a rapid screening method, does not provide insight into the morphology and internal organization of multicomponent samples such as nanoemulsions and nanoemulsion gels. Particular caution in interpreting results on droplet size and their distribution is required for semi-solid nanoemulgels since the DLS signal could be affected by aggregates of polymeric gelling agents [56]. This is probably the reason why preservation of nanodroplets after gelation is insufficiently discussed in the literature. Being aware of the limitations of dynamic light scattering, the complex structure of nanoemulsions and nanoemulsion gels with three different gelling agents was additionally investigated by Atomic Force Microscopy.

**Figure 6:**
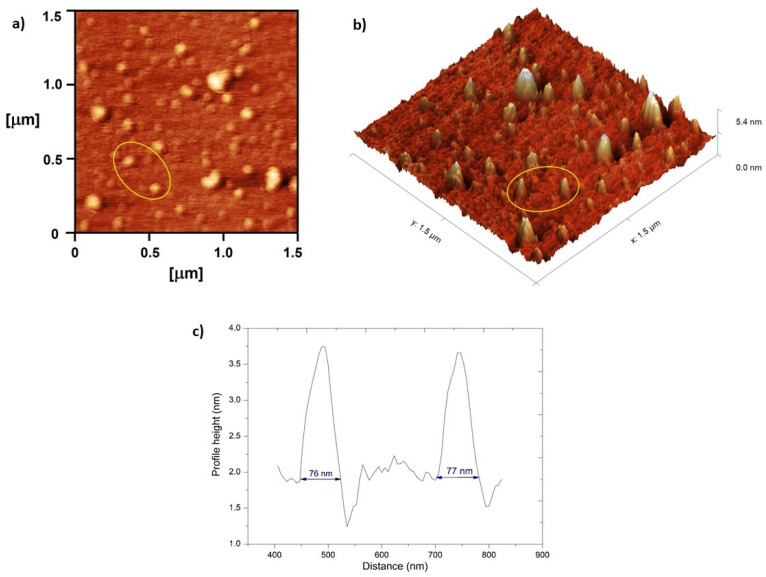
Atomic Force Microscopy images obtained for nanoemulsion NE_F1a prior gelation (1:500 *v*/*v*) on the surface area 1.5 × 1.5 µm^2^: (**a**) 2D topography, (**b**) 3D topography, and (**c**) profile height of two selected nanodroplets (marked in yellow circle).

The AFM analysis of the NE_F1a nanoemulsion sample on the imaging surface 1.5 × 1.5 µm^2^ confirmed the presence of small diameter droplets (<100 nm) of an evenly spherical shape, homogeneously distributed through the sample. Although the AFM analysis indicated very small diameter droplets (about 76 nm) (Figure 6c), this result differs slightly from the one obtained by the DLS method, where the average droplet size was even smaller (55.07 nm). However, it should be kept in mind that the height profiles that were analyzed matched medium-diameter droplets from a representative 2D topography and that numerous smaller droplets are visible on the 2D and 3D representations of topographies (Figure 6a,b), which is in good agreement with the DLS results. Furthermore, as previously mentioned, nanoemulsions are known to be sensitive systems, and the presence of some drops of a slightly larger diameter is expected and most likely the result of aggregation during sample preparation [57,58].

**Figure 7:**
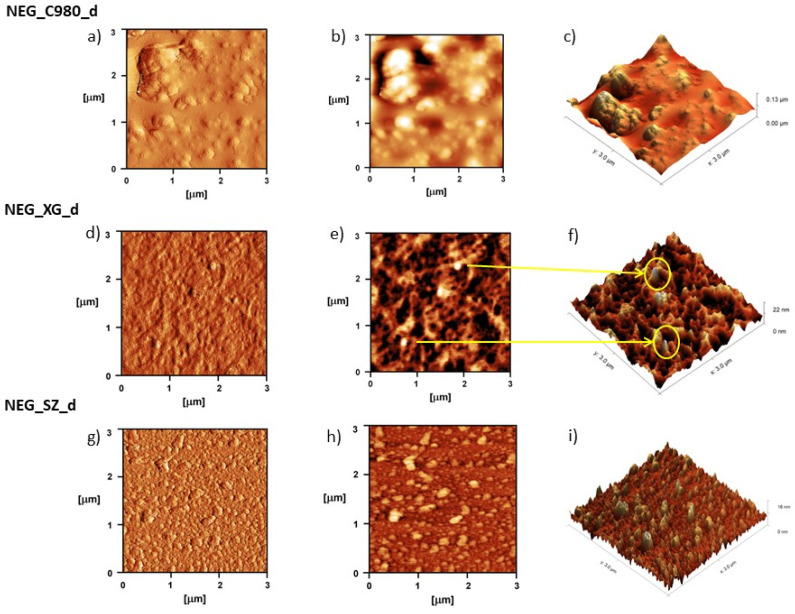
AFM analysis of nanoemulsion gels with: carbomer 980 (NEG_C980_d), xanthan gum (NEG_Xg_d), and polyacrylate crosspolymer-6 (NEG_SZ_d) (1:2000 *v*/*v*): (**a,d,g**) corresponding 2D error signals, (**b,e,h**) 2D topography, and (**c,f,i**) 3D topography.

The AFM analysis provided valuable insight into the morphology and internal organization of the directly gelled nanoemulsion gels (Figure 7). The 2D and 3D topographies on a 3 × 3 µm^2^ area of the sample NEG_C980_d clearly showed densely packed clusters of carbomer 980, particularly prominent in certain parts of the sample. This is probably a direct consequence of uneven neutralization of the polyacrylate chains with the trometamol during preparation. The large sample thickness (0.13 µm), as a result of the high density of this formulation, disabled a clear observation of small nanoemulsion droplets that are probably locked within the gel network. The micrographs of the sample NEG_XG_d indicated the extended, highly porous internal organization. The significantly lower sample height (22 nm) allowed for a clear observation of small-diameter nanodroplets that in shape and diameter resemble the droplets observed in the micrographs of the pure nanoemulsion. The formulation NEG_SZ_d with polyacrylate crosspolymer-6 showed compact organization, with neatly arranged, clearly visible droplets. The absence of large aggregates originating from the unswollen gelling agent and the preservation of nanodroplets after gelation demonstrate the inert nature and great potential of this innovative gelling agent to be used in this type of formulation.

The AFM analysis confirmed that direct gelation, although initially considered a more aggressive approach compared to the indirect method, did not lead to a disruption of the nanoemulsion structure, as confirmed by the presence of visible nanodroplets on the sample’s surface, particularly in the formulation with polyacrylate crosspolymer-6 and xanthan gum. To the best of our knowledge, this study shows the first successful use of AFM for visualization of the nanoemulsion gels’ internal structure.

#### 3.2.3. FT-IR and DSC Analysis of Nanoemulsion Gels

Next, complex interactions between the varied gel matrix and the nanoemulsion, as well as the overall nanodroplets preservation after direct vs. indirect gelling transformation, were analyzed with FT-IR and DSC.

**Figure 8:**
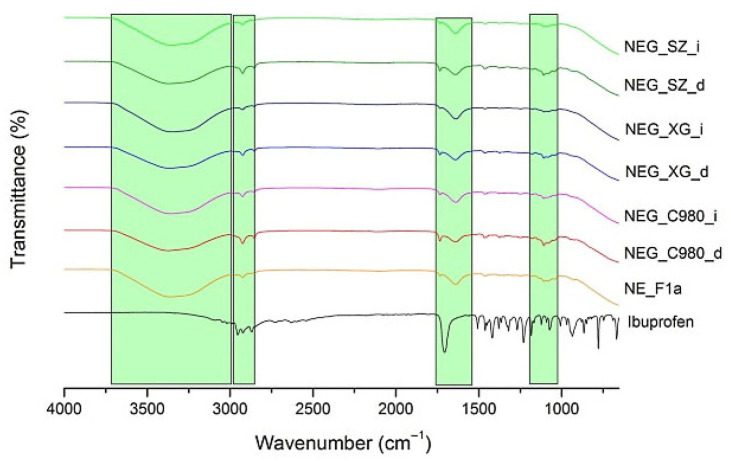
A comparative layout of the nanoemulsion gels’ FT-IR spectra relative to the ones of the nanoemulsion and pure model drug.

FT-IR analysis confirmed that gelation with all three tested gelling agents did not disrupt the nanoemulsion structure, neither via the direct nor indirect approach. All the characteristic peaks (between 2950 and 2870, 1700 and 1630, and 1200 and 1100 cm^−1^) originating from the nanoemulsion were preserved in the NEGs’ spectra (Figure 8). No additional peaks indicating the nanostructure degradation during transformation could be observed. However, it is important to emphasize that subtle differences were observed between gels obtained through direct and indirect gelation. The characteristic peaks exhibited higher intensity in all gels prepared via direct transformation, suggesting that this approach leads to stronger interactions between the gel network and the nanoemulsion compared to the indirect gelation method.

In all nanoemulsion-based gels, a broad peak between 3600 and 3200 cm^−1^ was observed, originating from free OH groups present in the surfactant and co-solvent molecules. Notably, all gels exhibited a less intensive peak compared to the spectrum of the nanoemulsion, suggesting the formation of hydrogen bonds between the gel network and the free OH groups of the nanoemulsion. This was most pronounced for the NEG_C980_d sample, indicating that direct gelation of the nanoemulsion with carbomer 980 leads to the strongest interactions between the 3D gel matrix and nanodroplets. This finding aligns with droplet size measurements, where the highest increase in droplet size after gelation was observed for NEG_C980_d. The peak between 2900 and 2800 cm^−1^, likely associated with the stretching of aliphatic C-H bonds from the oil phase molecules (Supplementary Material, Figure S3), remains preserved in all tested nanogels. The preservation of this peak suggests that the oily phase was successfully incorporated into the hydrophilic gel network without disrupting the nanoemulsion structure. The peak associated with C=O stretching vibrations from ibuprofen (1699 cm^−1^) remained present in all NEGs but significantly reduced in intensity compared to pure ibuprofen. This observation supports previous findings indicating that this small amphiphilic molecule is still localized within the stabilizer layer, closer to the aqueous phase, where it interacts with stabilizer molecules and co-solvents. Peaks corresponding to ether and alkoxide groups from free co-solvent and emulsifier molecules (1100 cm^−1^) were retained in all NEGs but slightly more pronounced in those obtained via the direct approach.

**Figure 9:**
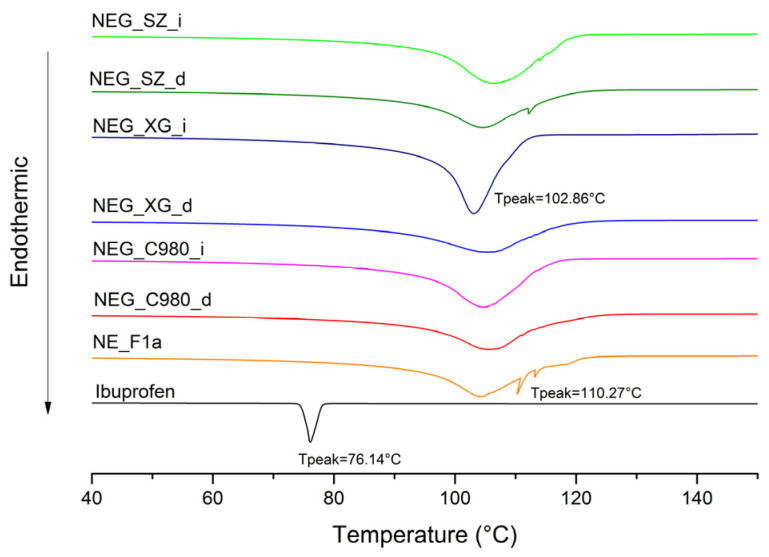
DSC scans of the investigated formulations.

DSC analysis supported the findings from FT-IR spectroscopy, highlighting distinct differences in the intensity of endothermic effects between NEGs obtained via direct vs. indirect gelation (Figure 9). The more pronounced endothermic peaks in directly transformed gels suggest stronger interactions with the gel network compared to those formed through indirect gelation. Similarly to nanoemulsions, the sharp endothermic peak near 76 °C characteristic of crystalline ibuprofen is no longer observed in the DSC thermograms of nanoemulsion-based gels. This confirms that even after transformation into the gel form, ibuprofen remains encapsulated within the stabilizing layer of nanodroplets, indicating that the gelation process did not disrupt the nanoemulsion structure. Minor endothermic peaks at 110.27 °C present in the sample NE_F1a thermograms, probably attributed to the evaporation of a small amount of bound water, were no longer detected in NEG thermograms. This finding supports the assumption that interactions between the nanoemulsion and the gel network are most likely mediated by hydrogen bonding. An exception was observed for the NEG_SZ_d formulation, where a small peak at this temperature remained visible, suggesting that this novel gelling agent forms a gel network in which nanodroplets are bound within the 3D gel matrix by other interactions as well. In the thermogram of the NEG_XG_i sample, a broad endothermic peak around 100 °C was observed. This effect is likely associated with the degradation of the gel network and release of a substantial amount of bound water, which may also be related to the lower viscosity of this system.

### 3.3. Integrating Rheological Characterization and Performance Testing into the Regulatory Translation of Innovative Formulations

As regulatory accepted [59], rheological behavior is a very important property of topical dosage forms that could reveal the microstructure organization of these drug delivery systems, affect certain manufacturing processes, and determine sensory properties. Drug release is also directly impacted by microstructure; it is well known that the rheological characteristics of pharmaceutical formulations intended for topical application influence the rate at which the active ingredients are released [60,61]. Nanoemulsion gels are complex structured delivery systems in which nanodroplets are immersed in the swollen gel network [22]. Given that the composition of nanoemulsion gels includes, on one hand, a hydrogel network, and on the other, a significant proportion of oils and surfactants, it is logical to assume that these innovative vehicles will also exhibit complex rheological behavior. Thus, to assess the effects of various gelling agents and the gelation method (direct vs. indirect approach) on the organization of the nanoemulgels’ microstructure, the rheological behavior was subsequently examined.

**Figure 10:**
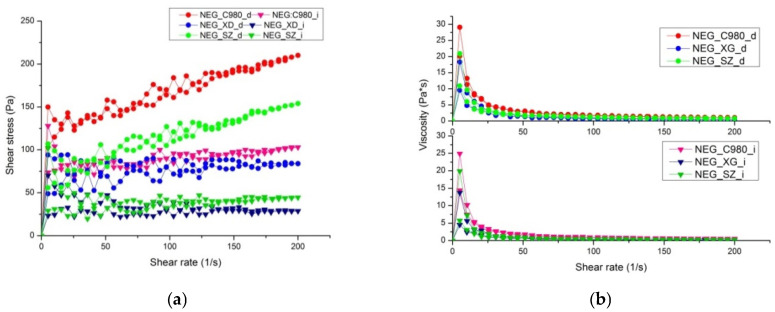
Flow curves (**a**) and viscosity vs. shear stress curves (**b**) of the developed nanoemulgels.

All formulations exhibit non-Newtonian, shear-thinning, thixotropic behavior, as evidenced by flow curves and critical rheological parameters (Figure 10 and Table 6). Investigated gels showed an increase in shear stress and a decrease in viscosity with an increase in shear rate. Similar rheological behavior of the nanoemulsion-loaded hydrogels was earlier reported in the literature [62]. Among the directly gelled formulations, NEG_C980_d demonstrated the highest shear stress values across the entire shear rate range, probably suggesting a more rigid network formation due to stronger interactions between the nanoemulsion and the gel matrix [63]. The NEG_SZ_d formulation also exhibited significant shear stress values, though lower than NEG_C980_d, indicating moderate structuring and possibly more favorable skin applicability. In contrast, NEG_XG_d had the lowest shear stress, suggesting a weaker gel network. For indirectly gelled formulations, a similar trend was observed but with consistently lower shear stress values compared to their direct counterparts. This indicates that indirect gelation leads to weaker interactions within the gel matrix, likely due to a less uniform incorporation of the nanoemulsion into the polymeric network.

The viscosity vs. shear rate profiles further supported these observations. All formulations exhibited an initial moderate viscosity that rapidly decreased with increasing shear rate, confirming their shear-thinning nature [64]. The directly gelled formulations (NEG_C980_d, NEG_SZ_d, and NEG_XG_d) retained higher viscosity values throughout the shear range, implying stronger structural integrity. The indirect gels (NEG_C980_i, NEG_SZ_i, and NEG_XG_i) showed a more pronounced drop in viscosity at lower shear rates, indicating lower resistance to flow and weaker gel structuring.

The rheological analysis confirmed that the gelation mechanism significantly influences the rheological properties of nanoemulsion gels. Direct gelation consistently resulted in robust gel networks, while indirect gelation produced somewhat weaker structures. Among the gelling agents, carbomer 980 formed NEGs the most resilient to the applied stress, which is reflected in the lowest hysteresis areas compared to other formulations, while xanthan gum exhibited the weakest structuring. These findings suggest that different gelling agents have diverse potential for the nanoemulsion entrapment by forming a variety of chain entanglements in the 3D gel structures. They correlate well with the AFM, DSC, and FT-IR results, reinforcing the role of molecular interactions in determining gel network microstructure.

Finally, performance of the developed NEGs was tested via in vitro ibuprofen release [59]. When assessing the microstructure of new topical dosage forms, it is of utmost importance to evaluate the drug release kinetics as well since complex vehicles are expected to impact all phases involved in the drug delivery to the target site of action [65,66]. Drug release mechanisms are known to be significantly influenced by the drug’s solubility in the carrier, droplet/particle size, and rheological characteristics. Thus, IVRT may be able to discern subtle variations among formulations and facilitate the selection of the best candidates for additional optimization. However, it should be kept in mind that IVRT provides only a preliminary insight into the kinetics of drug release in an in vitro environment influenced by the vehicle’s properties, while in vitro permeation (IVPT) and in vivo studies are necessary to explore complex interactions between the carrier, the drug, and the skin barrier [67,68].

**Figure 11:**
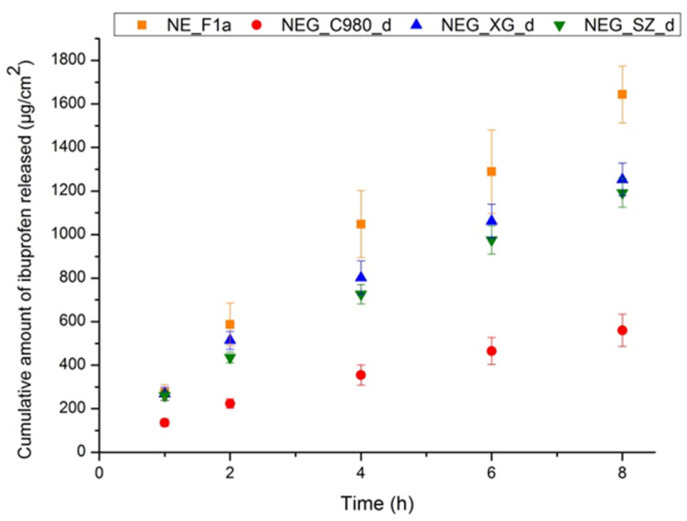
Cumulative amount of ibuprofen released from nanoemulsion and three different nanoemulgels during 8 h of experiment (see Supplementary Material, Figure S7 for comparative representation of cumulative % of ibuprofen released throughout 24 h).

In this study, IVRT was conducted to evaluate how differences in the nanoformulations’ microstructure affect the release rate/extent of ibuprofen. As represented in Figure 11, the release rates decreased in the following order: NE_F1a > NEG_XG_d > NEG_SZ_d > NEG_C980_d. The nanoemulsion showed the most rapid ibuprofen release, following the kinetics of the first order. This was in direct correlation with its low viscosity as well as the fact that the stabilizer layer was the only barrier for ibuprofen to circumvent prior to making contact with the diffusion membrane. On the other hand, nanoemulsion gels release ibuprofen with somewhat slower kinetics due to the higher viscosity of the continuous phase and the additional environmental barrier in the form of the gel network. By fitting the results through various mathematical models, it was apparent that the Korsmeyer–Peppas model best described the kinetics of ibuprofen release from nanoemulsion gels (Supplementary Material, Table S3), suggesting that due to their complex structure, the release mechanism is conditioned by a combination of polymer network relaxation and the diffusion of ibuprofen through it [69].

Differences in the 3D matrix organization among nanogels, identified by AFM, turned out to be a significant factor affecting the release of ibuprofen from nanoemulsion gels. Namely, the extended and porous structure of the gel in the case of the xanthan gum NEG showed release kinetics most similar to the nanoemulsion itself, indicating that after diffusion from the oil core, ibuprofen freely moved through the cavities of the gel network. Nanogel with polyacrylate crosspolymer-6 showed similar release rate kinetics as formulation NEG_XG_d. On the other hand, a densely packed matrix of nanoemulgel with carbomer 980 intensely slowed down the diffusion of ibuprofen, so the cumulative amount of released ibuprofen from this formulation after 8 h of experiment remained still very low.

In addition to internal organization, viscosity also appears to be a factor that greatly influenced the release profile, which turned out to be inversely correlated. Namely, sample NEG_C980_d showed the highest apparent viscosity (29.13 ± 1.55 Pa·s), which was another limiting factor for ibuprofen release, as the release rate was the slowest compared to other formulations. On the other hand, among nanogels, the fastest release was evidenced in the formulation that contains xanthan gum, which showed the lowest viscosity (18.37 ± 2.31 Pa·s) among the directly thickened nanoemulgels.

## 4. Conclusions

The developed nanoemulsions were successfully transformed to nanoemulsion gels as more user-friendly, semi-solid dosage forms. However, full disclosure of the structure pre- and post-transformation requires a comprehensive product characterization package. In this study, the combination of FT-IR and DSC revealed complex interactions, EPR discerned localization of the small-molecule model drug within the surfactants/co-solvents’ microenvironment, while AFM successfully visualized nanodroplets, with or without the presence of aggregates originating from the applied gelling agent. This work contributes to an in-depth understanding of the complex intact structure of nanoemulsion gels, where nanodroplets are dispersed, achieved by the proposed set of complementary physico-chemical and microscopic techniques. This integrated methodological approach, which demonstrated the preservation of nanodroplets, may stimulate further research and development of nanoemulsion gels and promote their faster translation to the market, in line with the recommendations of European and other regulatory authorities.

Unexpectedly, all the varied gelling agents supported direct approach in NEG preparation. Additionally, novel polyacrylate crosspolymer-6 favorably contributed to the electrostatic repulsion (ZP increase to favorable −30.5 mV), while providing an elegant-yet-robustly-structured gel network, with satisfactory ibuprofen release kinetics. Having in mind that the recent FDA’s Guidance for Industry entitled *Reformulating Drug Products That Contain Carbomers Manufactured With Benzene* is effectively omitting a number of carbomers from further use, novel gelling agents will be more than welcome to the pharmaceutical development design space.

## Figures and Tables

**Table 1:** Ibuprofen solubility in different oils, surfactants, and co-solvents.

Excipient	Ibuprofen Solubility (mg/mL)
IPM	65.94 ± 0.51
MCT	42.21 ± 0.28
Decyl oleate	38.12 ± 0.11
Octyldodecanol	43.41 ± 0.29
Tween 80	203.65 ± 1.32
Tween 60	166.63 ± 0.95
Ethanol	337.78 ± 0.81
Pg	189.41 ± 0.51
PEG 400	239.75 ± 0.85

**Table 2:** The precise composition of the investigated nanoemulsions with or without ibuprofen.

Formulation		Composition (% *w*/*w*)					
		Ibuprofen	IPM	Tween 80/Span 80 (3:1)	PEG 400	Pg	Water Up To
Placebo	NE_F1	/	10	10	5	5	100
	NE_F2	/	10	10	/	5	100
	NE_F3	/	10	10	5	/	100
Active	NE_F1a	2	10	10	5	5	100
	NE_F2a	2	10	10	/	5	100
	NE_F3a	2	10	10	5	/	100

**Table 3:** Physico-chemical properties of ‘placebo’ and ‘active’ nanoemulsions.

Formulation		Z-Ave (nm)	PDI	Zeta Potential (mV)	pH	Conductivity (µS/cm)
Placebo	NE_F1	80.56 ± 0.41 c	0.251 ± 0.016	−14.10 ± 1.20 a	6.00 ± 0.03	135.23 ± 0.42 c
	NE_F2	130.00 ± 0.51 c	0.184 ± 0.021	−26.70 ± 0.98	6.42 ± 0.02	128.13 ± 0.67 c
	NE_F3	108.40 ± 0.45 c	0.257 ± 0.003 a	−25.80 ± 0.70	5.84 ± 0.04	152.37 ± 0.78 c
Active	NE_F1a	55.07 ± 0.82 f	0.075 ± 0.022 f	−11.07 ± 0.38 f	5.11 ± 0.01 d	166.35 ± 0.91 f
	NE_F2a	54.35 ± 1.16 f	0.140 ± 0.019 f	−9.12 ± 0.49 f	5.13 ± 0.05 f	94.10 ± 0.26 f
	NE_F3a	60.38 ± 0.92 f	0.206 ± 0.006 f	−18.30 ± 0.72 f	4.96 ±0.03 f	113.40 ± 0.03 f

^a^ *p* < 0.05, ^c^ *p* < 0.001 differences between placebo formulations; ^d^ *p* < 0.05, ^f^ *p* < 0.001 when compared to the corresponding placebo formulation.

**Table 4:** EPR parameters for placebo and ibuprofen-loaded nanoemulsions, obtained with 5-DSA and 16-DSA spin probes.

Spin Probe	EPR Parameters	NE_F1	NE_F1a	NE_F2	NE_F2a	NE_F3	NE_F3a
5-DSA	τR (ns)	2.11 ± 0.03	2.06 ± 0.06	2.13 ± 0.10	1.82 ± 0.08	2.13 ± 0.09	1.90 ± 0.06
	S	0.09 ± 0.01	0.13 ± 0.04	0.14 ± 0.01	0.09 ± 0.01	0.11 ± 0.03	0.10 ± 0.02
	αN (×10^−4^ T)	13.98 ± 0.12	13.75 ± 0.43	13.15 ± 0.19	14.02 ± 0.1	13.71 ± 0.37	14.01 ± 0.15
16-DSA	τR (ns)	0.40 ± 0.06	0.37 ± 0.03	0.32 ± 0.06	0.38 ± 0.02	0.34 ± 0.01	0.38 ± 0.03
	S	0.03 ± 0.00	0.04 ± 0.00	0.04 ± 0.00	0.03 ± 0.01	0.03 ± 0.00	0.04 ± 0.00
	αN (×10^−4^ T)	14.72 ± 0.08	14.69 ± 0.08	14.66 ± 0.10	14.72 ± 0.06	14.64 ± 0.01	14.76 ± 0.06

**Table 5:** Nanoemulsion droplet size (Z-ave), polydispersity index (PDI), zeta potential (ZP), and pH after transformation into nanoemulsion gels via direct (_d) or indirect (_i) approach.

Gelling Agent	NEG Sample	Z-ave (nm)	PDI	ZP (mV)	pH
Carbopol 980	NEG_C980_d	94.03 ± 0.63 c,f	0.222 ± 0.008 b,f	−25.4 ± 2.9 e	5.71 ± 0.01 c,f
	NEG_C980_i	63.91 ± 0.37 c,f	0.164 ± 0.021 b,e	−27.9 ± 1.3 f	5.53 ± 0.01 c,f
Xanthan gum	NEG_XG_d	67.73 ± 1.15 f	0.195 ± 0.007 c,e	−20.6 ± 2.1 e	5.46 ± 0.03 c,f
	NEG_XG_i	65.54 ± 1.35 f	0.225 ± 0.008 c,f	−21.1 ± 0.9 f	5.65 ± 0.01 c,f
Polyacrylate Crosspolymer-6 (Sepimax Zen)	NEG_SZ_d	71.63 ± 1.13 c,f	0.134 ± 0.007 d	−30.5 ± 0.9 f	5.72 ± 0.02 f
	NEG_SZ_i	60.65 ± 0.84 c,f	0.126 ± 0.008 d	−26.3 ± 2.1 e	5.65 ± 0.05 f

^b^ *p* < 0.01, ^c^ *p* < 0.001 differences between direct and indirect method of preparation; ^d^ *p* < 0.05, ^e^ *p* < 0.01, ^f^ *p* < 0.001 compared to the nanoemulsion NE_F1a.

**Table 6:** Numerical values of key rheological parameters.

Formulations	Maximal Apparent Viscosity (Pa·s)	Minimal Apparent Viscosity (Pa·s)	Hysteresis Area (Pa/s)
NEG_C980_d	29.13 ± 1.55	1.04 ± 0.09	1322.64
NEG_XG_d	18.37 ± 2.31	0.42 ± 0.05	2483.91
NEG_SZ_d	20.87 ± 3.44	0.77 ± 0.04	1507.88
NEG_C980_i	24.97 ± 2.31	0.52 ± 0.03	1854.44
NEG_XG_i	13.73 ± 1.74	0.14 ± 0.01	1923.80
NEG_SZ_i	19.86 ± 1.94	0.22 ± 0.05	2036.27

## Data Availability

The original contributions presented in this study are included in the main manuscript text and the Supplementary Material. Further inquiries can be directed to the corresponding author.

## Supplementary Materials

The following supporting information can be downloaded at https://www.mdpi.com/article/10.3390/pharmaceutics17121532/s1, Table S1. Average droplet size, PDI and appearance of selected samples, initially and 24h after preparation, using different HLB stabilizers’ mixtures; Figure S1. EPR spectra of tested placebo and active nanoemulsion formulations with 5-DSA and 16-DSA spin probes; Figure S2. FT-IR spectra of binary (excipient + ibuprofen) and physical (placebo NE_F1 + ibuprofen) mixtures; Figure S3. FT-IR spectra of excipients and ibuprofen, used during nanoemulsions’ formulation development; Figure S4. DSC thermograms of the used excipients and their corresponding binary mixtures with ibuprofen: a) Isopropyl myristate; b) Tween 80; c) Span 80; d) Propylene glycol; e) PEG 400; f) Physical mixtures of placebo formulation NE_F1 and ibuprofen in 1:1 and 2:1 ratios; Figure S5. DSC thermograms of the placebo (NE_F2 and NE_F3) and active (NE_F2a and NE_F3a) nanoemulsions; Table S2. Additional test that confirmed robustness of ibuprofen-loaded nanoemulsions; Figure S6. Nanoemulsion gels produced with carbomer 980, xanthan gum and polyacrylate crosspolymer-6, by direct and indirect gelation method; Figure S7. 24 hours release profiles (%) of the nanoemulsion NE_F1a and corresponding nanoemulsion gels produced with carbomer 980, xanthan gum and polyacrylate crosspolymer-6; Table S3. Coefficient of correlation (R2) and diffusion release exponent (n) obtained by fitting the IVRT data of developed nanoformulations with different mathematical models.

## Abbreviations

The following abbreviations are used in this manuscript:

16-DSA	16-doxylstearic acid
2D	Two-dimensional
3D	Three-dimensional
5-DSA	5-doxylstearic acid
AFM	Atomic force micoscopy
DAD	Diode array detector
DLS	Dynamic light scattering
DSC	Differential scanning calorimetry
EC	Electrical conductivity
EPI	Emulsion phase inversion
EPR	Electron paramagnetic resonance
FDA	Food and Drug Administration
FT-IR	Fourier Transform Infrared spectroscopy
HLB	Hydrophilic–lipophilic balance
HPLC	High-performance liquid chromatography
HPMC	Hydroxypropyl Methylcellulose
IPM	Isopropyl myristate
IVPT	in vitro permeation test
IVRT	in vitro release test
MCT	Medium-chain triglycerides
NaCMC	Sodium Carboxymethyl Cellulose
NE	Nanoemulsion
NEG	Nanoemulsion gel, nanoemulgel, nanogel
NSAID	Nonsteroidal anti-inflammatory drug
PBS	Phosphate-buffered saline
PDI	Polydispersity index
PEG 400	Polyethylene glycol 400
Pg	Propylene glycol
PIT	Phase inversion temperature
rpm	Revolutions per minute
S	Order degree
S/CoS	Surfactant/co-solvent ratio
SOR	Surfactant-to-oil ratio
Span 80	Sorbitane oleate, Span^®^ 80
Tween 80	Polysorbate 80, Tween^®^ 80
Z-ave	Average droplet size
ZP	Zeta potential
αN	Isotropic hyperfine coupling constant
τR	Rotational correlation time
