# Supplementary material for: Interfacial Molecular Interactions as Determinants of Nanostructural Preservation in Ibuprofen-Loaded Nanoemulsions and Nanoemulsion Gels
Source: Pharmaceutics. 2025 Nov 28;17(12):1532. doi: 10.3390/pharmaceutics17121532 (PMC12736200; doi:10.3390/pharmaceutics17121532)
Supplement: Supplementary file 1 [file pharmaceutics-17-01532-s001.zip › pharmaceutics-3971855-supplementary.pdf]

*pharmaceutics*

MDPI

# Interfacial Molecular Interactions as Determinants of Nanostructural Preservation in Ibuprofen-Loaded Nanoemulsions and Nanoemulsion Gels

**Anđela Tošić, Danijela Randjelović, Branka Ivković, Ana Gledović, Tijana Stanković, Jelena Đoković, Vassiliki Papadimitriou, Tanja Ilić, Snežana D. Savić and Ivana Pantelić**

**Supplementary Materials**

*1. Preformulation Study*

After selecting the oily phase of the formulation, preformulation study was carried out to assess the capacity of the chosen surfactants to efficiently emulsify isopropyl myristate while forming nanosized droplets. The results of preformulation study are presented in Table S1.

**Table S1.** Average droplet size, PDI and appearance of selected samples, initially and 24h after preparation, using different HLB stabilizers' mixtures.

| Approximate HLB value of surfactant mixture | Surfactant/co-surfactant mixture | Z-ave (nm) | PDI | Appearance | Appearance after 24h |
|---|---|---|---|---|---|
| 15 | Tween 80 | 135.0 ± 3.1 | 0.167 ± 0.007 | White, emulsion like appearance | Phase separation |
| 14 | Tween 80/Span 80 (90:10) | 96.8 ± 1.4 | 0.189 ± 0.011 | White, opalescent with bluish shine | Formation of emulsion ring, no signs of phase separation |
| 13 | Tween 80/Span 80 (80:20) | 120.8 ± 1.9 | 0.191 ± 0.008 | White, opalescent with bluish shine | Formation of emulsion ring, no signs of phase separation |
| 12.5 | Tween80/Span80 (75:25) | 107.8 ± 1.9 | 0.201 ± 0.040 | Opalescent, with bluish shine | Opalescent with bluish shine |
| 11.5 | Tween80/Span 80 (65:45) | 128.4 ± 1.1 | 0.348 ± 0.057 | White, emulsion like appearance | Phase separation |

When Tween 80 was used as a surfactant alone, whitish dispersions of small droplet sizes (135.0 ± 3.1 nm) were obtained. This sample showed phase separation, suggesting that Tween 80 failed to effectively coat the oily droplets of isopropyl myristate, which showed a tendency to coalesce and resulted with the final phase separation 24 h after preparation. Therefore, it was decided to introduce a co-surfactant into the system that would potentially improve the coating of oil droplets by forming a more compact interlayer at the phase boundary. Sorbitan monooleate was selected, which, due to its complementary structure with Tween 80, proved to be a good choice in this type of formulation. Namely, together with Tween 80, it forms a denser stabilizing monolayer and thus has a favorable effect on the stability of the system. By measuring the droplet size and polydispersity index, it was determined that the addition of Span 80 favorably affects the physicochemical properties of the formulation by decreasing the particle size, so Span 80 was introduced to the surfactant mixture in different ratios, thus varying the HLB value of the

mixture itself. The HLB value of the surfactant mixture was calculated by using the following formula:

$$\text{HLB(mix)} = \sum(\text{Xi} \times \text{HLBi})$$

Although all tested mixtures resulted in the formulations with smaller droplet size compared to the formulation containing only Tween 80, the mixture with the HLB value of 12.5 stood out. This mixture remained stable for several days of monitoring, without phase separation or the formation of a (nano)emulsion ring on the vial during storage. Therefore, surfactant mixture of Tween 80/Span 80 in 75:25 ratio was selected for further research.

## 2. Electron Paramagnetic Resonance (EPR) Spectroscopy

EPR spectra obtained for placebo and ibuprofen-loaded nanoemulsions with 5-DSA and 16-DSA spin probes are represented in a Figure S1.

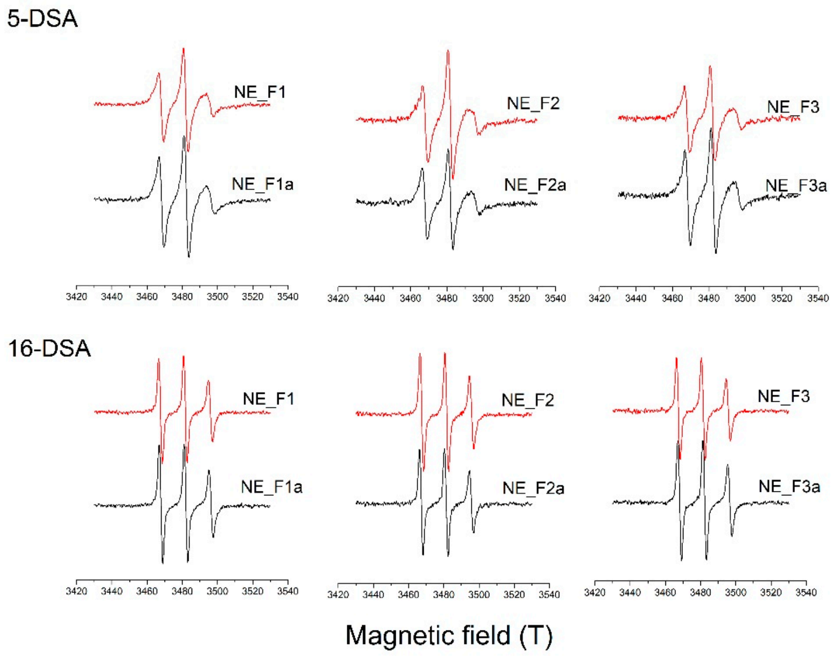


**Figure S1.** EPR spectra of tested placebo and active nanoemulsion formulations with 5-DSA and 16-DSA spin probes.

## 3. Fourier-Transform Infrared Spectroscopy (FT-IR)

~~Binary mixtures of ibuprofen and individual components of the nanoemulsion are represented, as well as the physical mixture of placebo nanoemulsion and ibuprofen are represented are analyzed by FT-IR spectroscopy and results are represented in the Figure S2.~~ The binary mixtures of ibuprofen with individual nanoemulsion components, along with the physical mixture of placebo nanoemulsion and ibuprofen, were analyzed using FT-IR spectroscopy, and the results are presented in a Figure S2. Given that characteristic peaks originating from ibuprofen are still present in all the spectra, it can be concluded that no covalent bonds were formed with any of the formulation ingredients. However, a reduction in the intensity of peaks originating from the carbonyl group of ibuprofen was observed in the spectra conducted from the mixture with Tween 80 and the mixture with

PEG 400, which confirmed assumption that ibuprofen is located within the stabilizer layer bound by hydrogen bonds with PEG 400 and Tween 80. Also, in the spectrum derived from the physical mixture of the placebo nanoemulsion and ibuprofen, clear characteristic peaks were observed which demonstrate ibuprofen presence in the undissolved form and in the water (external) phase of the nanoemulsion, but not within the nanodroplets. Absence of these peaks in the spectrum for NE_F1a formulation (Figure 2 in the main manuscript text), indicate that ibuprofen was incorporated within the nanoemulsion droplet in completely dispersed form.

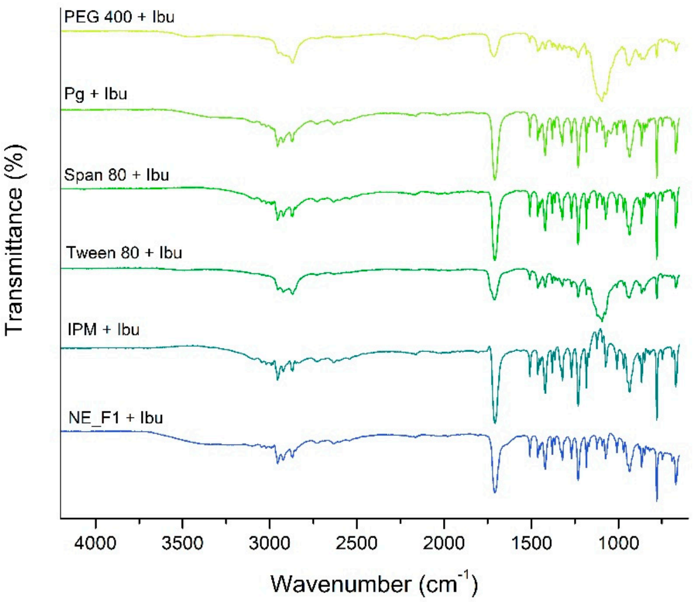


**Figure S2.** FT-IR spectra of binary (excipient + ibuprofen) and physical (placebo NE_F1 + ibuprofen) mixtures.

As shown in Figure S3, some of the excipients give characteristic peaks around 1725 $cm^{-1}$, due to the presence of the carbonyl group. Considering the presence of carbonyl group within ibuprofen structure as well, this part of the spectrum should be more closely observed. The stronger electron-donating (–I) effect of the alkoxy (–OR) substituents within the IPM, Tween 80, and Span 80 structures reduces the electrophilicity of the ester carbonyl group, which results in its absorption band appearing at a slightly higher wavenumber (around 1725 $cm^{-1}$). In contrast, the carbonyl group of ibuprofen is influenced by the opposing –I and +R effects of the adjacent hydroxyl group, leading to a decrease in its carbonyl stretching frequency. Consequently, the C=O band of ibuprofen is observed at a slightly lower wavenumber (around 1699 $cm^{-1}$), as clearly visible in the spectrum presented in Figure S3. Although similar, these peaks are sufficiently separated to allow the differences at these positions to be distinguished. In the placebo formulations (Figure 2, main text), small peaks can be observed near 1690 $cm^{-1}$, likely arising from the free carbonyl groups of Tween 80 and Span 80. Molecular interactions between stabilizers probably reduce the intensity of these peaks and shift them to lower wavelengths.

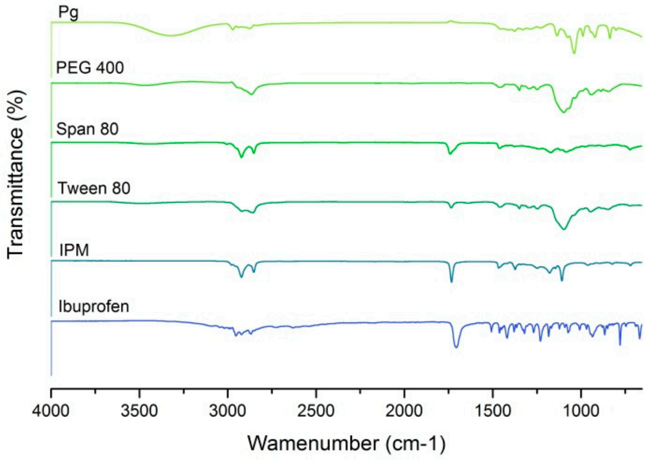


**Figure S3.** FT-IR spectra of excipients and ibuprofen, used during nanoemulsions' formulation development.

## 3. *Differential Scanning Calorimetry (DSC)*

DSC thermograms of all excipients included in the formulation and their corresponding binary mixtures with ibuprofen, as well as physical mixtures of the placebo formulation and ibuprofen in 1:1 and 1:2 ratios are shown in Figure S4. A broad endothermic peak is present in these additional samples between 40 and 80°C in almost all mixtures. These events suggest that ibuprofen is present in the liquid phase of these additional samples. Given the high solubility of ibuprofen in the surfactants and co-solvents, it is partly dispersed in them and partly present in the crystalline form, which was demonstrated with endothermic events in the thermograms. However, given the clear differences in the thermograms between the samples in which ibuprofen was physically added to the placebo formulation (Figure S4f) and the sample where ibuprofen was incorporated within the oily nanodroplet (Figure 3), the results suggest that in the case of the NE_F1a formulation, ibuprofen is localized within the nanodroplet in a fully solubilized form, in which no undissolved ibuprofen can be observed.

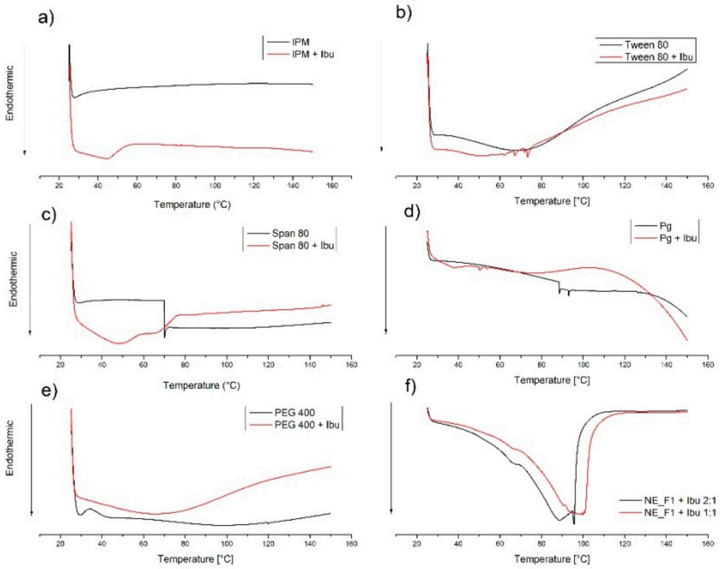


**Figure S4.** DSC thermograms of the used excipients and their corresponding binary mixtures with ibuprofen: (a) Isopropyl myristate; (b) Tween 80; (c) Span 80; (d) Propylene glycol; (e) PEG 400; (f) Physical mixtures of placebo formulation NE_F1 and ibuprofen in 1:1 and 2:1 ratios.

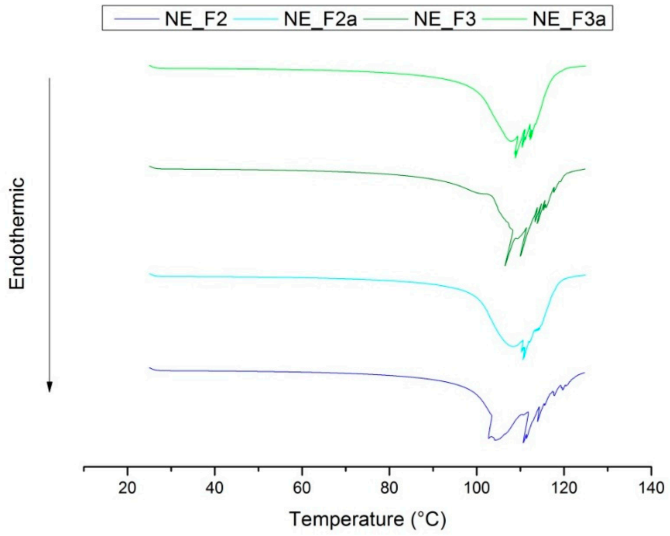


**Figure S5.** DSC thermograms of the placebo (NE_F2 and NE_F3) and active (NE_F2a and NE_F3a) nanoemulsions.

*Investigation of Robustness of Ibuprofen-Loaded Nanoemulsion*

The preliminary stability of the formulation was assessed by centrifugation test (4500 rcf for 90 min), dilution robustness test (1:100, 1:500 and 1:1000 dilution with ultrapure water) and quantification of drug content by validated HPLC method, and the results are represented in Table S2. The findings indicate that all three formulations remained stable after centrifugation (results were similar to the ones obtained by initial measurements, represented in Table 3 of the main manuscript text), as well as that they show high robustness, even after 1:1000 dilution. Drug content was in an acceptable range for all three formulations (between 90.85 and 100.5%). On the other hand, formulations did not pass the

heating-cooling test (40°C - room temperature - 4°C, in three repeated cycles), since an increase in the average droplet size was observed probably due to temperature-induced changes in surfactant packing and oil phase expansion. These results confirm that the developed formulations are indeed nanoemulsions, since by definition nanoemulsions represent kinetically stable but thermodynamically unstable systems, i.e., they could resist high kinetic stress, but may still undergo structural changes under extreme thermodynamic stress such as repeated heating–cooling cycles

**Table S2.** Additional test that confirmed robustness of ibuprofen-loaded nanoemulsions.

| | After centrifugation on 4500 rcf for 90 min | | Robustness to dilution 1:500 | | Robustness to dilution 1:1000 | | Drug content (%) |
|---|---|---|---|---|---|---|---|
| | Z-ave | PDI | Z-ave | PDI | Z-ave | PDI | |
| NE_F1a | 54.78 ± 0.45 | 0.129 ± 0.010 | 54.42 ± 0.37 | 0.091 ± 0.009 | 54.67 ± 0.90 | 0.062 ± 0.008 | 100.50 ± 0.74 |
| NE_F2a | 50.31 ± 1.13 | 0.182 ± 0.012 | 46.56 ± 0.37 | 0.115 ± 0.019 | 46.22 ± 0.81 | 0.099 ± 0.006 | 90.85 ± 2.59 |
| NE_F3a | 59.82 ± 0.70 | 0.239 ± 0.020 | 58.80 ± 1.02 | 0.210 ± 0.010 | 58.66 ± 0.52 | 0.183 ± 0.025 | 92.02 ± 7.82 |

## *4. Transformation of Nanoemulsions into Nanoemulsion Gels*

Nanoemulsions are transformed into nanoemulsion gels by direct and indirect method, and their visual appearances are represented in the Figure S6. All gels had a bluish shine, implying that the nanostructure was preserved within the gel network. Additionally, the most transparent gels were observed in the case of xanthan gum, while significantly denser gels were obtained by direct gelation.

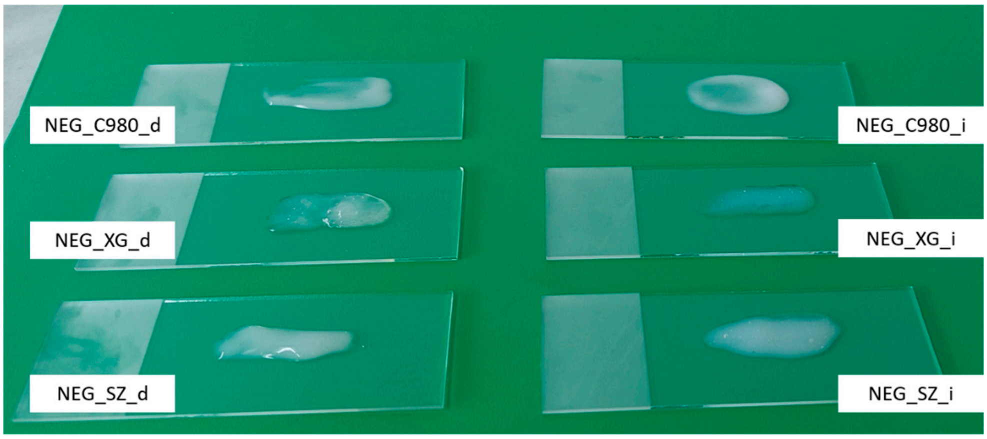


**Figure S6.** Nanoemulsion gels produced with carbomer 980, xanthan gum and polyacrylate crosspolymer-6, by direct and indirect gelation method.

## *5. In Vitro Release Testing*

Concerning the presentation of IVRT results, for the main manuscript text we have applied the recommendation given by the latest EMA's scientific guideline entitled "Quality and equivalence of locally applied, locally acting cutaneous products", i.e. that the quantity of active substance released in mass units per unit area at a given time should be reported. However, for direct comparison, it may be useful to present results in the form of cumulative % of released ibuprofen, especially when the release profiles are monitored for a longer period of time, as provided in Figure S7.

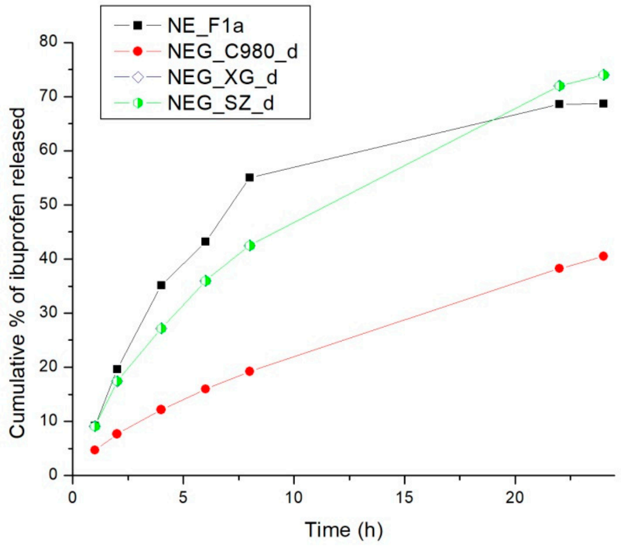


**Figure S7.** 24 hours release profiles (%) of the nanoemulsion NE_F1a and corresponding nanoemulsion gels produced with carbomer 980, xanthan gum and polyacrylate crosspolymer-6.

**Table S3.** Coefficient of correlation ($R^2$) and diffusion release exponent (n) obtained by fitting the IVRT data of developed nanoformulations with different mathematical models.

| | Zero order | First order | Higuchi | Korsmeyer-Peppas |
|---|---|---|---|---|
| NE_F1a | 0.9435 | 0.9965 | 0.9059 | 0.9825 (n=0.81) |
| NEG_C980_d | 0.8845 | 0.9193 | 0.9535 | 0.9995 (n=0.68) |
| NEG_XG_d | 0.8946 | 0.9650 | 0.9436 | 0.9950 (n=0.92) |
| NEG_SZ_d | 0.9336 | 0.9808 | 0.9291 | 0.9914 (n=0.99) |
